# Identification and classification of the genomes of novel microviruses in poultry slaughterhouse

**DOI:** 10.3389/fmicb.2024.1393153

**Published:** 2024-05-02

**Authors:** Keming Xie, Benfu Lin, Xinyu Sun, Peng Zhu, Chang Liu, Guangfeng Liu, Xudong Cao, Jingqi Pan, Suiping Qiu, Xiaoqi Yuan, Mengshi Liang, Jingzhe Jiang, Lihong Yuan

**Affiliations:** ^1^School of Life Sciences and Biopharmaceutics, Guangdong Pharmaceutical University, Guangzhou, Guangdong, China; ^2^Key Laboratory of South China Sea Fishery Resources Exploitation and Utilization, Ministry of Agriculture and Rural Affairs, South China Sea Fisheries Research Institute, Chinese Academy of Fishery Sciences, Guangzhou, Guangdong, China; ^3^Huadu District Animal Health Supervision Institution, Guangzhou, Guangdong, China; ^4^College of Marine Ecology and Environment, Shanghai Ocean University, Shanghai, China; ^5^Department of Chemical and Biological Engineering, University of Ottawa, Ottawa, ON, Canada

**Keywords:** poultry slaughterhouse, microviruses, genome, clustering, host

## Abstract

Microviridae is a family of phages with circular ssDNA genomes and they are widely found in various environments and organisms. In this study, virome techniques were employed to explore potential members of Microviridae in a poultry slaughterhouse, leading to the identification of 98 novel and complete microvirus genomes. Using a similarity clustering network classification approach, these viruses were found to belong to at least 6 new subfamilies within Microviridae and 3 higher-level taxonomic units. Genome size, GC content and genome structure of these new taxa showed evident regularities, validating the rationality of our classification method. Our method can divide microviruses into about 45 additional detailed clusters, which may serve as a new standard for classifying Microviridae members. Furthermore, by addressing the scarcity of host information for microviruses, the current study significantly broadened their host range and discovered over 20 possible new hosts, including important pathogenic bacteria such as *Helicobacter pylori* and *Vibrio cholerae*, as well as different taxa demonstrated different host specificities. The findings of this study effectively expand the diversity of the Microviridae family, providing new insights for their classification and identification. Additionally, it offers a novel perspective for monitoring and controlling pathogenic microorganisms in poultry slaughterhouse environments.

## 1 Introduction

Slaughterhouses are an essential pathway for livestock and poultry meat products to move from farms to consumers’ tables. They are also key points for the transmission of pathogenic microorganisms (Benfu et al., 2021). Due to the high density and mobility of poultry when entering the market or slaughterhouses, poultry comes from diverse sources and has varying hygienic conditions, and may carry multiple pathogenic microorganisms (Große-Peclum et al., 2023). The slaughter process is prone to contaminating the environment and the personnel involved. Additionally, the waste generated during poultry slaughter and processing further provides favorable conditions for the proliferation of pathogenic microorganisms (Wu et al., 2019). The interactions between animals, the environment, and occupational personnel form a closed-loop microbial transmission chain. Some pathogenic microorganisms can infect occupational personnel through direct contact, while others may have an indirect impact by contaminating the environment. Existing research indicates that the detection rate of certain pathogenic microorganisms, such as *Campylobacter* and *Salmonella* and avian influenza viruses, is significantly higher among occupational personnel compared to the general population (Arzey et al., 2012; García-Sánchez et al., 2019; Zhang et al., 2019; Rasschaert et al., 2020; Zeng et al., 2021). Therefore, conducting extensive microbial research at the interface of animals, the environment, and occupational personnel in poultry slaughterhouses is of significant importance. China is a major player in livestock and poultry farming and consumption. China’s total meat consumption is nearly 100 million tons per year, accounting for 27% of the global total. In 2022, the domestic meat production reached 92.27 million tons, with poultry meat contributing 24.43 million tons, constituting 26.5% of the total global meat production (National Bureau of Statistics, 2023).

Bacteriophages, a type of viruses that specifically infect bacteria, are the most abundant life forms on earth (Díaz-Muñoz and Koskella, 2014). It is estimated that there are as many as 10^31^ virus particles on earth (Geoghegan and Holmes, 2017; Mushegian, 2020), representing a vast and largely untapped reservoir of biological resources. The presence of pathogens in slaughterhouses creates favorable conditions for the survival of bacteriophages. As a result, investigating the diversity, types, and hosts of bacteriophages in poultry slaughterhouses can enhance our understanding of the composition, transmission, and the interplay between pathogens and bacteriophages in such a unique environment. In addition, occupational personnel are at the core of operations, and exposures to pathogenic bacteria increase the risk of infections of this particular group of population, which is a major public health safety hazard. Therefore, it would be advantageous to fully explore and develop potential functional phage species based on the high diversity of phages in poultry slaughterhouses in order to effectively purify the environment, and block the spread of pathogenic bacteria in poultry slaughterhouses to safeguard public health safety.

Members of the Microviridae family are one of the most widely distributed single-stranded DNA viruses and their natural hosts include pathogenic bacteria such as *Spiroplasma*, *Chlamydia*, and Enterobacteria (2012). Recent studies suggest the importance of the Microviridae family on the virosphere (Roux et al., 2012). At present, the only Microviridae subfamilies recognized by International Committee on Taxonomy of Viruses (ICTV) are Bullavirinae and Gokushovirinae (Walker et al., 2021), which cannot fully reflect the diversity of viruses in this family. While more Microviridae subfamilies, such as Alpavirinae (Krupovic and Forterre, 2011) and Pichovirinae (Roux et al., 2012) have been proposed, the number of classified groups and host information about the Microviridae family remain severely limited in the literature. This study takes the unique and biologically significant environment of a poultry slaughterhouse in Guangzhou, Guangdong Province, China, and employs both multiple displacement amplification (MDA) method (Lasken, 2009) and metagenomics sequencing to obtain environmental virus sequencing data from the poultry slaughterhouse (DSV, Dataset of Slaughterhouse Virome) in Guangzhou. Within this dataset, we discovered a diverse set of novel viruses belonging to the Microviridae family. A detailed analysis of 98 nearly complete Microviridae genomes revealed their classification into at least six new subfamilies and three higher-level taxonomic units. Comparative analysis with publicly available viral databases demonstrated the high resolution of this classification. Additionally, more than 20 potential hosts for microviruses were identified. This study expands our knowledge of the evolution, diversity, and host range of the Microviridae family, providing insights into the potential biosecurity and ecological significance of these microviruses in poultry slaughterhouses.

## 2 Materials and methods

### 2.1 Sample collection

A total of three types of samples were collected from a poultry slaughterhouse in a district of Guangzhou: animals, occupational personnel, and environmental samples. The environmental samples included air, soil, sludge, swabs from transportation vehicles, and swabs from the slaughterhouse workshop. The collection protocols were as follows: (1) Animal Samples: Sterile cotton swabs were inserted into the oral cavity and cloaca of chickens or ducks, rotated three times, and then removed. The swab’s tail was discarded, and the swab was immersed in sterile 0.5% BSA-PBS buffer for preservation. Three chickens or ducks from each of the three spaces (caged area, pre-slaughter area, slaughter area) had their oral and cloacal swabs mixed to form one sample. (2) Occupational Personnel Nasal Swab Samples: To collect nasal swab samples from occupational personnel, a sterile cotton swab was gently inserted into the nasal pharynx of the participating volunteer. After a few seconds, the swab was gently rotated and removed. The swab’s tail was discarded, and the swab was immersed in sterile 0.5% BSA-PBS buffer for preservation. Nasal swab samples from a single person with both nostrils were placed in an individual sample collection tube. Written informed consent was obtained from all participants. (3) Air Samples: BioSamplers KIT (225-9595, SKC, Eighty Four, PA) were installed at approximately 1.5 meters above floor at the ventilation points in the slaughter area, pre-slaughter area, and caged area (3 sampling points in total). Using 0.5% BSA-PBS buffer at a flow rate of 8 mL/h, sampling was conducted for 12 h per day at 110 V. Each day’s collection was considered one air sample, and this process was repeated continuously for 3 days. The collected samples were stored in PBS buffer. (4) Soil Samples: Soil samples were collected using the quincunx sampling method at various spaces (Xiao et al., 2023), including the entrance of the poultry slaughterhouse, the slaughter workshop, and the pre-slaughter caged area. Each sample weighed 5–10 g. (5) Sludge Samples: Sludge samples were collected at the four corners of the sewage discharge pool, with approximately 10 mL of sewage collected per sample. (6) Environmental Swab Samples: Sterile cotton swabs were used to collect environmental samples from the slaughter workshop, pre-slaughter area, caged area, and poultry transportation vehicles. Five swab samples were collected from each space or vehicle, discarding the swab tails and placing them in sterile 0.5% BSA-PBS buffer for preservation (Table 1). After collection, all samples were stored at 4°C, transported to the laboratory in a cooler, and then stored long-term at −80°C in an ultra-low-temperature freezer. This study was approved by the Medical Ethics Committee of the School of Public Health, Sun Yat-sen University (Permit No. [2018] No. 001).

**Table 1:** Sample information.

Sample classification		Source	Sample quantity	Sample pool names (Abbreviations)	Sequencing number
Animal		Chicken	90	SC	C-SXAO
		Duck	60	SD	D-SXAO
Human		Occupational Personnel	20	SN	CD-SXO-S
ENV^1^	Air	Caged Area	3	AC	CD-SXA-1
		Pre-slaughter Area	3	AW	CD-SXA-2
		Slaughter Area	3	AS	CD-SXA-3
		Soil	12	Soil	CD-SXG
		Sludge	4	Sludge	CD-SXD
	Swab	Transport Vehicle	15	ST	CD-SXt
		Slaughterhouse Workshop	35	SS	CD-SXS

1 ENV, Environment.

### 2.2 Sample pool preparation

In order to analyze the virus content and types in samples from different spaces (i.e., slaughter area, pre-slaughter area, caged area) and different types (i.e., air, animals, sludge.) within the slaughterhouse, we combined samples of the same type collected from the same space to prepare sample pools: (1) Combined oral and cloacal swabs from 20 ducks in each of the three spaces (caged area, pre-slaughter area, slaughter area) to create a pool (total of 60 ducks). Combined oral and cloacal swabs from 30 chickens in each of the three spaces to create a pool (total of 90 chickens). (2) Combined nasal swab samples from 20 frontline slaughterhouse workers into one pool. (3) Combined air samples collected continuously for 3 days from each sampling point, creating one pool per sampling point. (4) Combined soil samples collected from each space (mixed samples with four or more points) into one pool. (5) Combined sludge samples collected from each sewage discharge pool into one pool. (6) Combined swab samples from seven slaughterhouse process points in the workshop into one pool. (7) Combined swab samples collected from three poultry transportation vehicles into one pool. Sample pool information is provided in Supplementary Table S2.

### 2.3 Virus enrichment, nucleic acid extraction and amplification

Virus-like particles (VLPs) were enriched separately based on the different properties of the samples. Approximately 0.4 g of sludge and soil samples were added to about 2–5 volumes of sterile SB buffer (0.2 M NaCl, 50 mM Tris–HCl, 5 mM CaCl2, 5 mM MgCl2, pH 7.5). For air samples and swab samples, they were directly added to 2–5 volumes of sterile SB buffer to dissolve the virus particles. After three cycles of freeze-thawing, the particles were completely resuspended in 10 times the volume of pre-chilled SB buffer. All samples were centrifuged at 1,000, 3,000, 5,000, 8,000, 10,000, and 12,000 × *g* for 5 min at 4°C using a Sigma 3 K30 centrifuge (Sigma Laborzentrifugen GmbH, Germany), and the supernatant was collected. Subsequently, the supernatant was filtered through 0.22 μm Millipore filters (Burlington, MA) to further remove any cell debris and organelles. The filtrate was transferred to 28% sucrose solution and ultra centrifuged at 300,000 × *g* for 2 h in a Himac CP 100WX ultracentrifuge (Hitachi, Tokyo, Japan). The supernatant was discarded, and the pellet was re-suspended in 720 μL of water, 90 μL of 10 × DNase I Buffer, and 90 μL of DNase I (1 U/μl) (TAKARA, Japan). The suspension was thoroughly re-suspended, incubated at 37°C with shaking for 60 min, stored overnight at 4°C, and then transferred to a 2 mL centrifuge tube.

Total nucleic acids were extracted using the HP Virus DNA/RNA Kit (R6873; Omega Bio-Tek, Norcross, USA), and carrier RNA was not used during the process to avoid potential interference with sequencing results. The concentration of RNA was quantified using the Qubit^™^ dsDNA HS Assay Kit (Q32851) and Qubit™ RNA HS Assay Kit (Q32855) (Thermo Fisher Scientific, Waltham, USA).

Virome research heavily relies on amplification, as the viral biomass in natural samples is often very low. Due to variations in most amplification methods, quantitative studies of viral data present challenges at present (Hosono et al., 2003; Pan et al., 2013). In the current study, uniform genome amplification (WGA) and transcriptome amplification (WTA) were performed using the repi-g Cell WGA and WTA Kit (150,052, Qiagen, Hilden, Germany), based on the multiple displacement amplification (MDA) method (Lasken, 2009; Picher et al., 2016; Stepanauskas et al., 2017; Tithi et al., 2018). Compared to other amplification methods, MDA has several significant advantages. It can replicate fragments up to 70 kb, provides more uniform genome coverage, and has a fidelity 1,000 times higher than Taq polymerase amplification. Most importantly, MDA has the capability to preferentially amplify circular ssDNA genomes, which is crucial for the study of viruses in the Microviridae (Paez et al., 2004; Roux et al., 2016; Wei et al., 2018).

### 2.4 Library construction and sequencing

The amplified DNA was quantified using gel electrophoresis and Nanodrop 2000 spectrophotometer (Thermo Fisher Scientific, Waltham, MA). Ultrasonic random shearing (Covaris M220) was performed to generate fragments with lengths ≤800 bp. Fragment ends were repaired using T4 DNA Polymerase (M4211, Promega, Madison, Wisconsin), Klenow DNA Polymerase (KP810250, Epicentre, Madison, Wisconsin), and T4 Polynucleotide Kinase (EK0031, Thermo Fisher Scientific, Waltham, MA). Fragments in the range of 300–800 bp were collected after electrophoresis. After amplification, the libraries were pooled, and paired-end sequencing of 150 bp, 250 bp, or 300 bp was performed on the Novaseq 6000, HiSeq X ten, and Miseq platforms (Illumina, San Diego, California) (Patch et al., 2018; Ravi et al., 2018; Modi et al., 2021).

### 2.5 *De novo* assembly, annotation, and sequence filtering

All samples underwent virome sequencing, resulting in approximately 700 million raw sequencing reads. High-quality clean reads were generated using Fastp v0.20.0 (Chen et al., 2018) (options: --correction, --trim_poly_g, --trim_poly_x, --overrepresentation_analysis, --trim_front1 = 16, --trim_tail1 = 2, and --length_required = 50). Reads matching the Illumina sequencing adapters were removed (option: –detect_adapter_for_pe). The clean reads in libraries that were in the same assembly group were pooled and assembled using MEGAHIT v1.2.9 (Li et al., 2015) with the default settings. Contigs shorter than 500 bp were discarded. To detect low-abundance contigs, clean reads not mapped to contigs from the initial assembly were reassembled for two additional rounds, and all remaining reads were merged and assembled together. Contigs from all four assembly rounds were pooled and clustered at 97% global average nucleotide identity with at least 90% overlap of the shorter contig using Cd-hit-est v4.8.1 (Li and Godzik, 2006) (options: -aS 0.9 -c 0.97 -G 1 -M 0 -T 0 -g 1), resulting in 169,216 nonredundant contigs.

Diamond (Buchfink et al., 2015), an advanced sequence annotation tool known for its high accuracy and speed, was employed. BLAST searches of the NCBI NR database against non-redundant protein sequences significantly reduce the number of false positive results compared to BLAST searches against virus databases only (Nouri et al., 2018; Yao et al., 2020). However, BLAST accuracy decreases for short fragments and cannot be used for dissimilar sequences (Jiang et al., 2011). Nevertheless, combining multiple viral mining tools such as CheckV (Nayfach et al., 2021) and VirSorter2 (Guo et al., 2021) improves the success rate of predicting unknown viral sequences. Identifying and classifying putative viral sequences is challenging due to a lack of reliable annotations (Handley and Virgin, 2019). Given these challenges, we used Diamond v0.9.24.125 (options: -e 1e-10, --max-target-seqs 50), for annotation against the NCBI NR database.

CheckV (version 0.7.0) and its associated databases (Nayfach et al., 2021) were used to assess the completeness of assigned viral genomes. After removing false-positive contigs that matched more host genes than viral genes, 1,224 nearly complete viral genomes were obtained. Diamond were used to determine the taxonomy of the viral contigs at the family level. Diamond annotations were further processed in MEGAN6 (Huson et al., 2016) using default parameters with two scripts (daa2rma and rma2info) and parsed into taxonomic annotations. A total of 98 viral sequences (Contig IDs are shown in Supplementary Table S1) were identified as complete genomes and annotated as belonging to the Microviridae for further in-depth analysis.

### 2.6 Open reading frame (ORF) prediction and alignment

Cenote-Taker 2 (Tisza et al., 2020) was used in the current study to predict open reading frames (ORFs) in 98 virus genomes (use default parameters, add ‘-am True’ at the end of the command line to output genome map files). Cenote-Taker 2 is a virus discovery and annotation tool that can be used on the command line. It utilizes a highly sensitive model of signature viral genes to identify familiar or novel virus sequences from the user’s input of contigs. Cenote-Taker 2 performs better in discovering virus sequences in complex datasets, with lower false positive and false negative rates compared to similar tools (Tisza et al., 2020). The major capsid protein or capsid protein sequence (major capsid protein is preferred if available, otherwise capsid protein is chosen. These proteins are collectively referred to as “Cap”) was selected from the predicted results of each viral sequence. NCBI BLASTP (Altschul et al., 1990, 1997) was used to compare ORF sequences with the NR database, with an Expect threshold (*e*-value) set to 10^−5^. For each ORF in the alignment results, the top ten protein sequences with their complete genomic sequences were downloaded based on identity. Duplicate sequences were removed from all downloaded sequences. SnapGene^1^ was utilized to open the Cenote-Taker2 output file for visualizing the genomic structure.

### 2.7 Sequence similarity clustering analysis and genomic feature statistics

Cap sequences predicted for DSV microvirus were collected, along with top 10 ranked Cap sequences from the aforementioned BLASTP results, and introduced 20 Cap sequences from microviruses that have been definitively classified by the ICTV. A total of 577 sequences were aligned with each other using DIAMOND (Buchfink et al., 2015) (version 0.9.14.115, options: --evalue = 0.00001) to build a matrix of sequence similarities. Based on the Score value output by DIAMOND alignment, Gephi (Bastian et al., 2009) (version 0.9.7, Layout selected as Fruchterman Reingold, Rescale weight = True, with other parameters set to default) was used to construct a clustering network graph [appropriate score values were selected based on clustering effect to form clear clusters as demonstrated in previous studies (Jiang et al., 2023; Zhu et al., 2024)]. The nodes were colored on different sequence sources, hosts, or virus classification results. Furthermore, the Cap sequences used by Kirchberger et al. (2022) were integrated with the above data. The same method was employed to construct a similarity clustering network graph and color it, aiming to compare the network clustering resolution of our research method with that of Kirchberger et al.

We further performed statistical analysis on the genome length and GC content of each cluster. Statistical analysis was performed by one-way ANOVA and Turkey test at a significant level of *p* < 0.05.

### 2.8 Host prediction

hostG and cherry are software tools specifically designed for predicting bacteriophage hosts, and they are considered superior to existing virus host prediction software (Shang and Sun, 2021; Shang and Sun, 2022; Howell et al., 2024). All complete genome sequences included in the analysis in section 2.7 were subjected to host prediction using hostG (Shang and Sun, 2021) (output results taking genus, genus_score > 0.7) and cherry (Shang and Sun, 2022) (output results taking Top_1_label, Score_1 > 0.7), analyzing the relationship between these viruses and their hosts, as well as the proportion of these hosts.

### 2.9 Phylogenetic tree based on cap sequences

Cap is a conserved gene of microviruses (Quaiser et al., 2015) with approximately 500 amino acids in length, and is commonly used as a phylogenetic marker for the classification of evolutionary branches or subfamilies within the Microviridae. Multiple sequence alignment was performed using MAFFT (Katoh and Standley, 2013) ambiguous regions were removed using TrimAl (Capella-Gutiérrez et al., 2009), and a maximum likelihood phylogenetic tree based on Cap sequences was constructed using IQtree (Minh et al., 2020) (version 2.1.4). ModelFinder (Kalyaanamoorthy et al., 2017) was set to MFP (for ModelFinder Plus), and 1,000 ultrafast bootstrap replicates were used. The tree was visualized using iTOL (Letunic and Bork, 2016) (version 6.5.2).^2^

### 2.10 Principles of classification and naming of viral sequences

**Figure 1:**
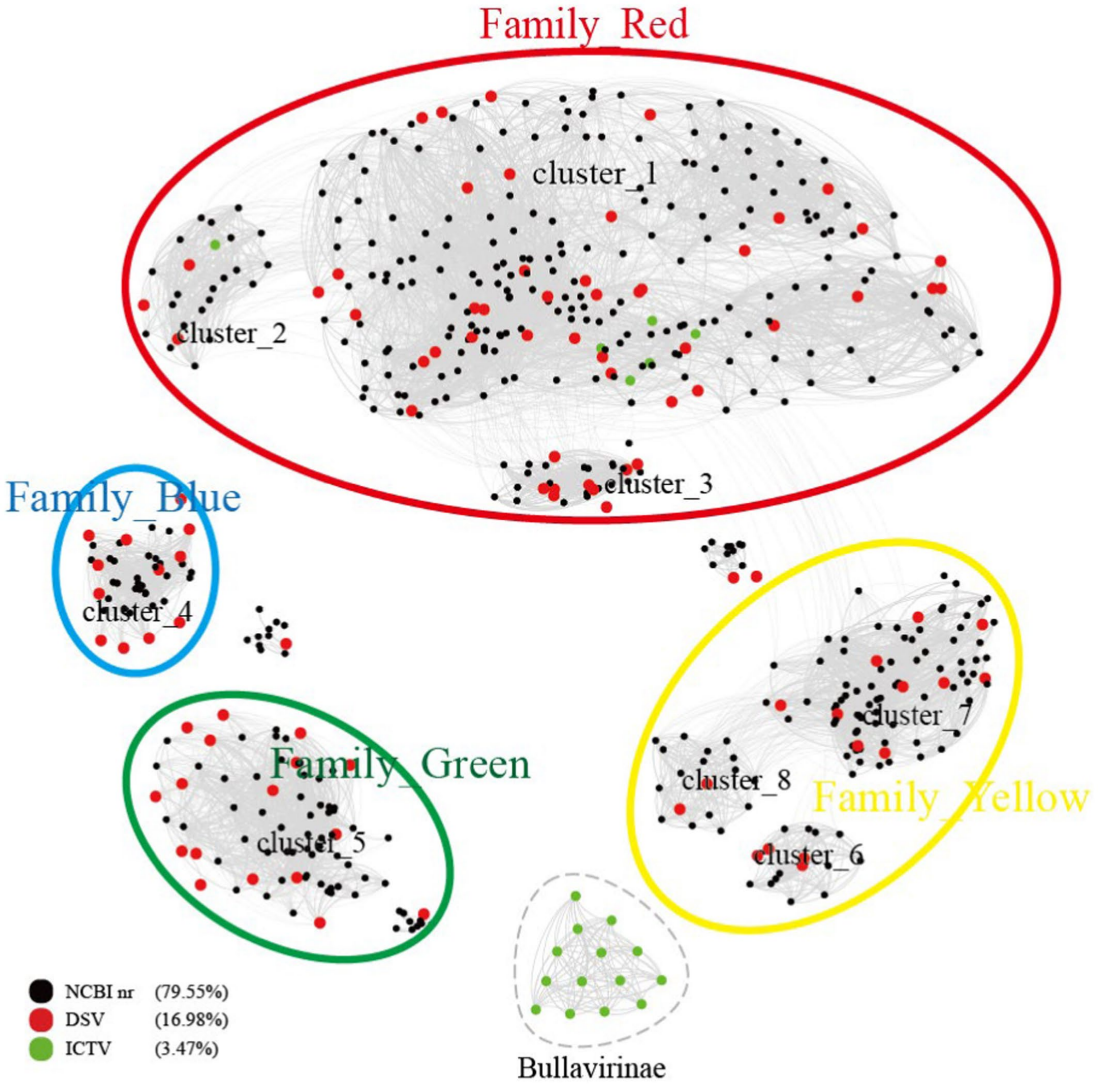
Similarity clustering network of Cap of microviruses from poultry slaughterhouses and related microvirus groups. The network includes identified microvirus Cap sequences from the DSV data (*n* = 98), along with related Cap sequences from the NR data (*n* = 459) and microvirus Cap sequences from the ICTV data (*n* = 20). The similarity clustering network was constructed using Gephi (version 0.9.7) based on Diamond (version 0.9.14.115) alignment score. Gray connections represent Diamond Blastp score > 480.

According to the clustering in Figure 1 and cherry host prediction results, DSV-related viral sequences are named, respectively. Taking cluster_1 as an example, if a sequence has host prediction results, it is named based on the host, such as the contig sequences CD-SXS-WGA-1-k141_397009 and CD-SXD-WGA-1-k141_230904 are named Bdellovibrio microvirus C1_1 and Bdellovibrio microvirus C1_2, respectively. Similarly, CD-SXG-WGA-1-k141_33139 and CD-SXG-WGA-1-k141_32996 are named Escherichia microvirus C1_1 and Escherichia microvirus C1_2. If the sequence has no host prediction results, contig sequences like CD-SXD-SXG-WGA-all--k141_113185 and CD-SXD-SXG-WGA-all--k141_328845 are named DSV microvirus C1_1 and DSV microvirus C1_2, and so forth. The original sequence ID and their corresponding names are listed in Supplementary Table S1.

## 3 Results

### 3.1 Discovery of novel subfamilies of Microviridae

According to the ICTV standards, Microviridae includes two subfamilies (Bullavirinae and Gokushovirinae) and seven described genera (Walker et al., 2021). Among them, the subfamily Bullavirinae has three genera, comprising 14 species. The subfamily Gokushovirinae has four genera, consisting of 8 species. We selected 98 complete DNA viral genomes from the DSV dataset, annotated as Microviridae, with a genome integrity exceeding 90%, for in-depth analysis. All DSV genomes have lengths ranging from 4 to 6 kb, consistent with the genome size of Microviridae (2012). As predicted, these viruses all have Cap with lengths of 450–600 amino acids (AA). According to the Cap similarity clustering network graph (Figure 1), the Microviridae sequences from DSV, along with the related sequences aligned in NR and the Microviridae sequences from ICTV (totaling 577 sequences), roughly cluster into 9 clusters (cluster_1 to 8 and Bullavirinae). Among them, 14 Bullavirinae sequences recognized by ICTV formed a separate cluster (light green). The remaining 6 ICTV- recognized Gokushovirinae sequences are distributed in cluster_1 (C1) and cluster_2 (C2). According to this classification criterion, C1 and C2 should belong to the Gokushovirinae. While the other 6 clusters (cluster_3 to 8) do not contain ICTV sequences, indicating they may represent new subfamilies within the Microviridae.

Based on the similarity of these 9 clusters, we can divide them into 5 major clusters, tentatively referred to as Family_Red, Family_Blue, Family_Green, Family_Yellow, and the independent Bullavirinae cluster. Family_Red contains 6 sequences recognized by the ICTV as Gokushovirinae; therefore, we tentatively equate Family_Red with the Gokushovirinae. If using Bullavirinae as the standard, then the separate cluster represents at least one subfamily level, while a major cluster formed by multiple clusters may represent a higher level of classification. However, using Gokushovirinae as the standard, one subfamily can be distributed in different clusters (Family_Red). As for whether the major clusters of Family_Blue, Family_Green, and Family_Yellow should be regarded as new subfamilies or elevated to new families requires further discussion.

### 3.2 Expanding the potential hosts of microviruses

**Figure 2:**
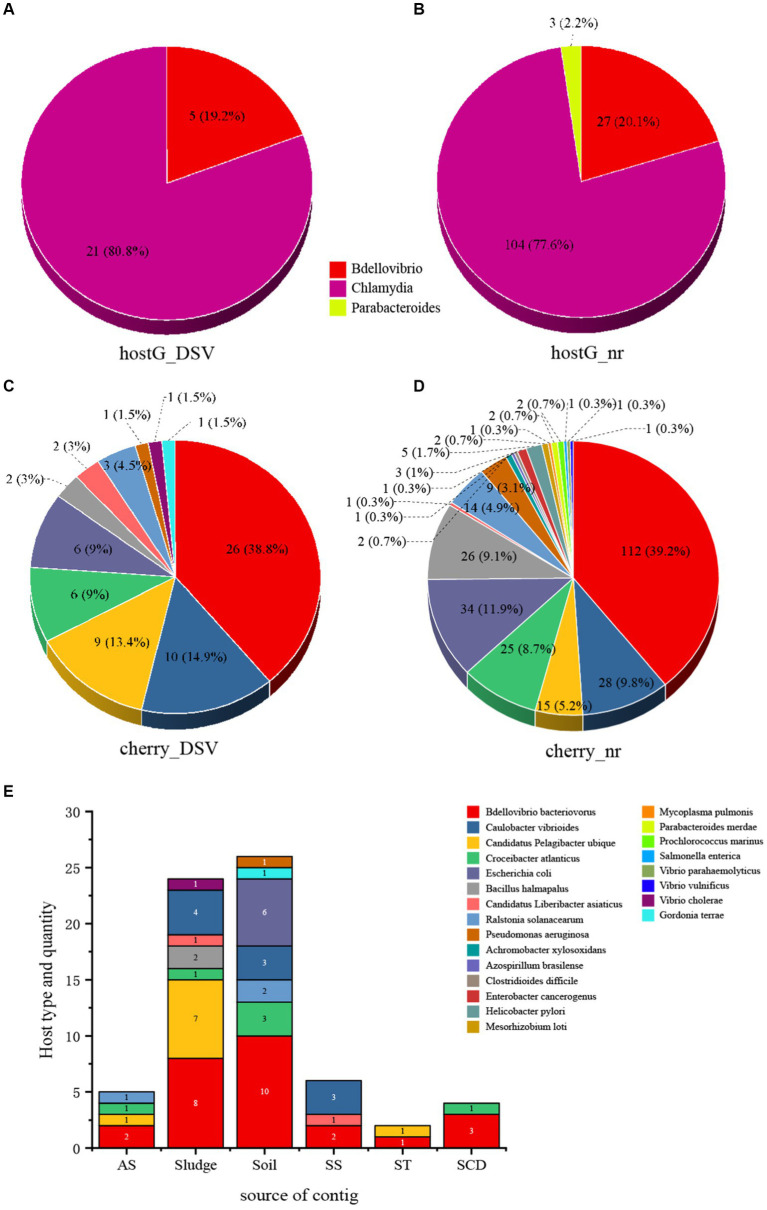
The host types and quantity statistics of microviruses from poultry slaughterhouses and related groups. **(A)** hostG (Shang and Sun, 2021) results of DSV sequences. **(B)** hostG results of NR sequences. **(C)** Cherry (Shang and Sun, 2022) results of DSV sequences. **(D)** Cherry results of NR sequences. Score > 0.7. **(E)** Host types and quantity predicted by cherry for corresponding DSV sequences. AS (All soil and sludge of slaughterhouse); Soil (Soil of slaughterhouse); Sludge (Sludge of slaughterhouse); SS (Swab of slaughterhouse workshop); ST (Swab of poultry transport vehicle); SCD (Oral and cloacal swabs of chickens and ducks).

Host prediction was performed on the 98 newly discovered microvirus sequences from this study, along with their associated 459 NR sequences and 20 ICTV sequences, using hostG (Shang and Sun, 2021) and cherry (Shang and Sun, 2022). In the hostG results, only NC_002643.1 from ICTV was accurately predicted to have a host (*Bdellovibrio*). However, in the cherry results, the majority of hosts were consistent with the ICTV results, indicating that the success rate and accuracy of cherry predictions were higher than hostG. According to the hostG results, the main hosts for DSV were *Bdellovibrio* and *Chlamydia* (Figures 2A,B). For NR sequences, the hosts were mainly distributed in the *Bdellovibrio*, *Chlamydia*, and *Parabacteroides*. Although these results align well with the current understanding of microvirus hosts, results from cherry suggest (Figures 2C,D) that the hosts of microviruses may be far more diverse than these three genera.

Although *Bdellovibrio bacteriovorus* and *Escherichia coli* are the predominant hosts in the NR-derived virus hosts, respectively. Cherry also predicted hosts such as *Caulobacter vibrioides* and *Bacillus halmapalus*, indicating numerous microvirus hosts that have not been previously reported. Among the DSV-derived virus hosts, *Bdellovibrio bacteriovorus* still dominated, followed by *Caulobacter vibrioides* and *Candidatus Pelagibacter ubique*, representing novel hosts. In addition, NR data revealed the presence of human and animal pathogens such as *Helicobacter pylori* and *Enterobacter cancerogenus*. The DSV data also identified potential hosts including *Vibrio cholerae* and *Pseudomonas aeruginosa*. This suggests that the host range of microviruses within the Microviridae may be extensive, and that there are likely more potential hosts yet to be discovered.

From the perspective of sample types, the highest abundance of microviruses was observed in soil and sludge samples, corresponding to a higher diversity and quantity of their respective hosts (Figure 2E). *Bdellovibrio bacteriovorus*, as a typical host for microviruses, showed a higher proportion across various samples. *Caulobacter vibrioides* also exhibited high abundance in sludge, soil, and the slaughterhouse workshop (Figure 2E). While *Escherichia coli*, *Gordonia terrae*, and *Pseudomonas aeruginosa* were predicted only in soil samples, *Vibrio cholerae* was exclusively found in sludge samples. Other host bacteria were detected across different sample types. This indicates a close relationship between the detection of microviruses and the distribution of their host bacteria, displaying certain characteristics in various sample types.

### 3.3 Genome length and GC content

**Figure 3:**
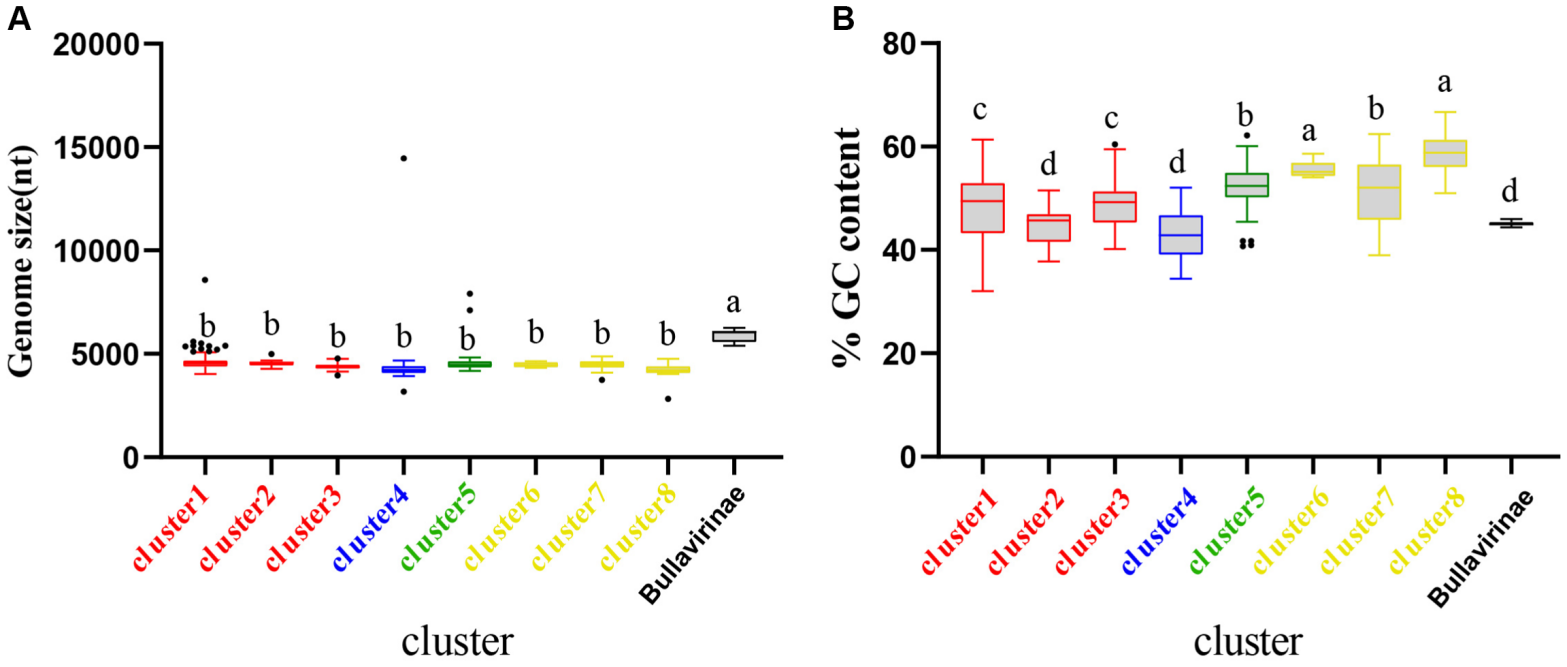
Genomic features of each cluster of microviruses from poultry slaughterhouses and related groups. **(A)** Distribution of microviruses genome sizes in each cluster from Result 3.1. **(B)** Distribution of microviruses genome %GC content in each cluster from Result 3.1. Red, blue, green, yellow, and black correspond to Family_Red, Family_Blue, Family_Green, Family_Yellow, and Bullavirinae, respectively. Turkey’s test was used, where *p* < 0.05 indicates significant differences, and *p* > 0.05 indicates no significant differences. In the group where the maximum mean value is located, mark it with the letter “a.” Then, compare this mean value with the mean values of other groups one by one. If there is no significant difference, label them with the same letter “a.” Continue this process until encountering a mean value with a significant difference, then label it with the letter “b.” Subsequently, use “b” as the standard for further comparisons. Repeat this process, labeling consecutive mean values with the letters “b” until encountering a mean value with a significant difference, which is then labeled as the letter “c.” This pattern continues for subsequent comparisons. The plot displays median values, 25th and 75th percentiles, 1.5 interquartile ranges, and outlier data points.

The genome sizes and GC content of viruses within the same family or genus are usually relatively consistent (Roux et al., 2019; Yuan et al., 2022). Based on the identification of 9 clusters in the previous sections, we further created boxplots illustrating their genome size and GC content (Figure 3). Both genome size and GC% exhibited high consistency within each of the 9 clusters, while significant differences were observed among different clusters. For instance, Bullavirinae showed distinct genome sizes and GC content compared to other groups. Individual scattered black dots outside the boxes in the figure represent sequences from the NR data. It has been reported that there is an association between the GC content of viral genomes and that of the hosts (Ha and Denver, 2018; Yuan et al., 2022). The differences in GC content between clusters may imply host-specific characteristics of each group. This phenomenon may be due to the close evolutionary history of members within each group, which has not led to significant host crossings or changes in genomic features. This similarity will also be observed in the subsequent host analysis of each cluster. These results indicate that the genome characteristics of microviruses from different taxonomic groups exhibit good consistency and indirectly validate the reliability of our classification method based on the similarity clustering network graph.

### 3.4 Phylogenetic analysis based on cap sequences

To better illustrate the diversity of DSV-related microviruses and their evolutionary origins, phylogenetic trees were constructed for each cluster based on the results in Figure 1. The sequences of DSV-related microviruses were classified and named according to the sample source and host type of the viruses (see Materials for reference).

**Figure 4:**
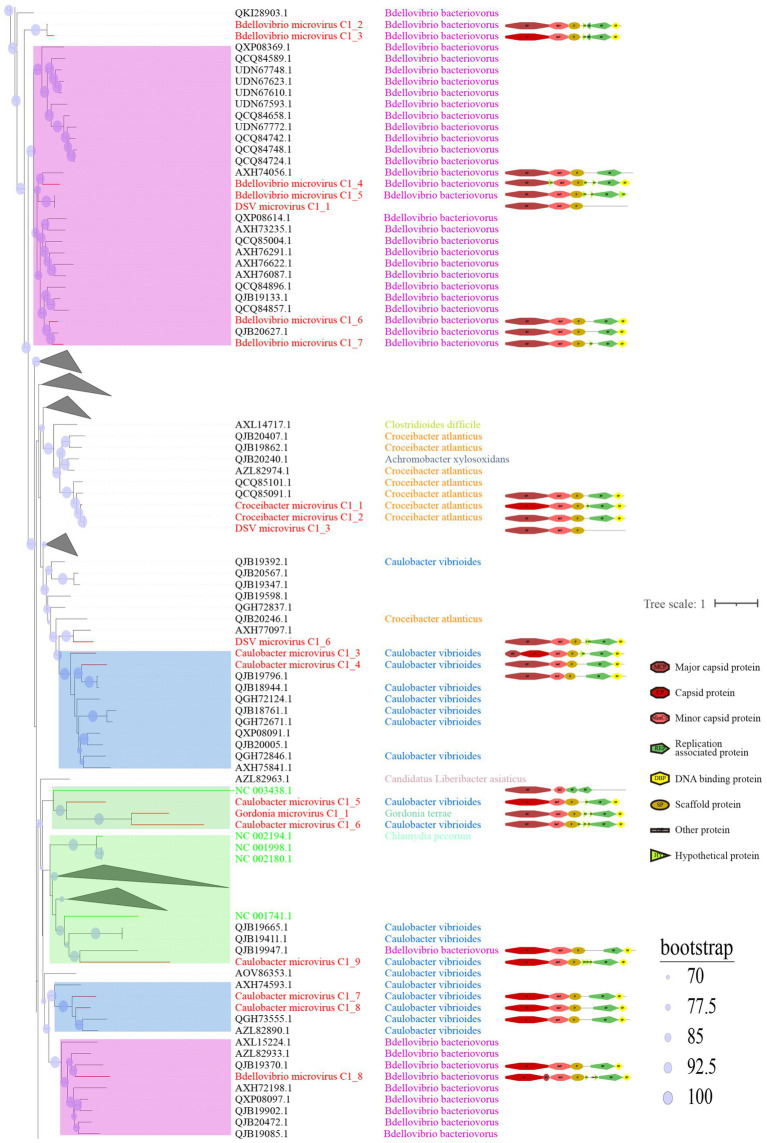
Phylogenetic tree, hosts, and genomic structure of cluster_1 microviruses from poultry slaughterhouse and related sources. The maximum likelihood phylogenetic tree was constructed based on the Cap sequences of Microviruses using IQtree (version 2.1.4). ModelFinder was set to MFP, and 1,000 ultrafast bootstrap replicates were performed, displaying bootstrap values >70. The red branches represent DSV microvirus sequences, green branches represent ICTV sequences, and black branches represent NR sequences. The third column shows host annotations predicted by Cherry, and the fourth column displays partial genomic structure diagrams.

C1 is the cluster with the highest number of members among the eight clusters and exhibits the most diverse range of host sources (Figure 4). Displayed are partial positions of the phylogenetic tree. Some phylogenetic branches have been collapsed, the complete phylogenetic tree is detailed in Supplementary Figure S1. Notably, in addition to typical hosts such as *Escherichia coli* and *Bdellovibrio bacteriovorus*, this cluster has hosts that were previously unreported, such as *Caulobacter vibrioides*, *Pseudomonas aeruginosa*, and *Helicobacter pylori*. *Caulobacter vibrioides* is a Gram-negative oligotrophic bacterium widely distributed in freshwater lakes and streams, serving as an important model organism for studying cell cycle regulation, asymmetric cell division, and cell differentiation (Abraham et al., 1999). *Pseudomonas aeruginosa* is a common multidrug-resistant pathogen, characterized by its capsule, Gram-negative nature, and aerobic or facultatively anaerobic growth, causing diseases in plants and animals, including humans (Diggle and Whiteley, 2020). *Helicobacter pylori* is a Gram-negative, flagellated spiral bacterium, classified as a class I carcinogen, responsible for approximately 89% of gastric cancer cases and associated with 5.5% of cancer cases worldwide (Violeta Filip et al., 2018; de Brito et al., 2019; Marghalani et al., 2020). In general, hosts within the same clustering branch are relatively homogeneous. For example, in Figure 4, the hosts in the purple-colored block branch are primarily *Bdellovibrio bacteriovorus,* while the hosts in the deep blue-colored block branch are mainly *Caulobacter vibrioides*.

From the sample sources perspective, DSV viruses in this cluster mainly originate from soil (CD-SXG) and sludge (CD-SXD), with a few from swab samples taken in the slaughterhouse workshop environment (CD-SXS) (Supplementary Table S1). In comparison, the sources of NR viruses are more diverse, including animal metagenomes, wastewater metagenomes, human metagenomes, and blackflies. As seen in the genomic structure diagram in Figure 4, members of C1 typically possess signature genes such as Major capsid protein or capsid protein (Cap), and Replication associated protein (Rep). Moreover, the genomes in this cluster often exhibit a sequential arrangement of Cap, Minor capsid protein (MinCP), Scaffold protein (SP), Rep, and DNA binding protein (DBP). However, a few viruses in this cluster have genome organization sequences that deviate from this pattern, such as DSV microvirus C1_4. Furthermore, Replication-associated protein was not predicted in DSV microvirus C1_1 and DSV microvirus C1_3. Coincidentally, these two sequences also lack host prediction results, likely suggesting the novelty of these viral genomes. Overall, sequences with closer phylogenetic relationships tend to exhibit more apparent consistency in host specificity and genomic structure.

**Figure 5:**
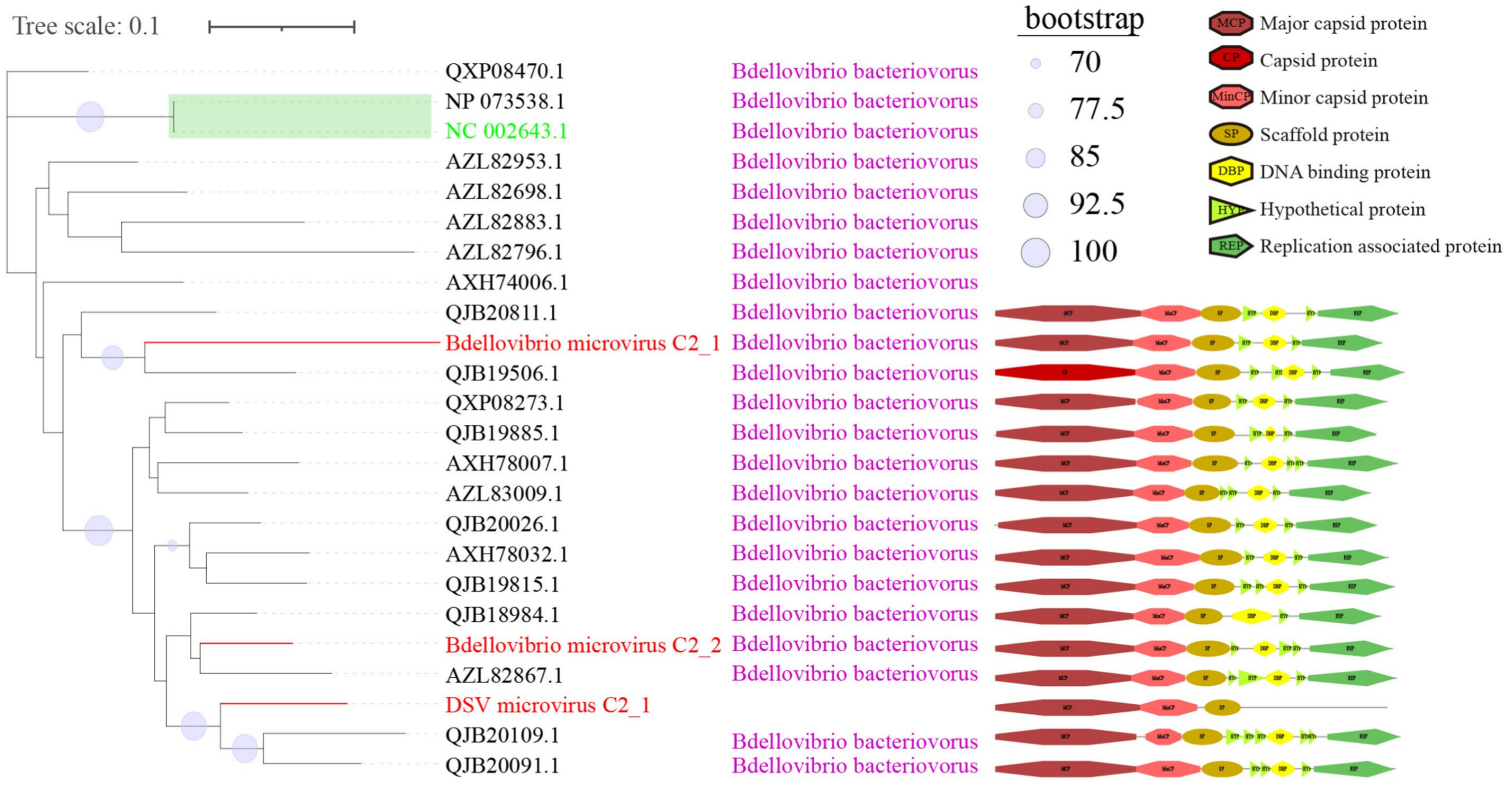
Phylogenetic tree, hosts, and genomic structure of cluster_2 microviruses from poultry slaughterhouse and related sources. The maximum likelihood phylogenetic tree was constructed based on the Cap sequences of Microviruses using IQtree (version 2.1.4). ModelFinder was set to MFP, and 1,000 ultrafast bootstrap replicates were performed, displaying bootstrap values >70. The red branches represent DSV microvirus sequences, green branches represent ICTV sequences, and black branches represent NR sequences. The third column shows host annotations predicted by Cherry, and the fourth column displays partial genomic structure diagrams.

C2 is a small viral cluster with a consistent host source, all being *Bdellovibrio bacteriovorus*, and a highly consistent genomic structure (Figure 5). The viruses in this cluster exhibit a sequential arrangement of Cap, MinCP, SP, DBP, and Rep, with Hypothetical protein (HYP) inserted on either side of DBP. Bdellovibrio microvirus C2_2 and Bdellovibrio microvirus C2_1 are closely related to AZL82867.1 and QJB19506.1, respectively. The genome of AZL82867.1 is derived from Honey bees, while QJB19506.1 originates from wastewater metagenome. This observation suggests the widespread presence of microviruses and their *Bdellovibrio bacteriovorus* hosts in various environmental settings. Only three sequences in cluster_3 (C3) were predicted to have hosts (Supplementary Figure S2), indicating that this group lacks sufficient host information and is a relatively novel group compared to C1 and C2. The predicted hosts for these three sequences are *Azospirillum brasilense* (*A. brasilense*) and *Enterobacter cancerogenus* (*E. cancerogenus*). *A. brasilense* is a microaerophilic nitrogen-fixing bacterium widely present in the rhizosphere worldwide, promoting plant growth (Tarrand et al., 1978; Tien et al., 1979). *E. cancerogenus* is a significant pathogen commonly found in human clinical specimens such as blood and cerebrospinal fluid. It is not sensitive to penicillin and cephalosporin (Farmer et al., 1985), and exploring bacteriophage targeting such multidrug-resistant pathogens is meaningful for developing phage therapy methods.

In the phylogenetic tree of C1 (Figure 4), the two green boxes belong to the same level of evolutionary branch. The ICTV sequence NC_003438.1 belongs to Spiromicrovirus, located in the branch of the first green box. The four ICTV sequences NC_002194.1, NC_001998.1, NC_002180.1, NC_001741.1 belong to Chlamydiamicrovirus, located in the branch of the second green box. It can be inferred that the branches at this level belong to members of the same genus. The C2 (Figure 5) group follows the same logic, NP_073538.1 and the ICTV sequence NC_002643.1 are in the same level evolutionary branch, likely also belonging to Bdellomicrovirus. According to this criterion, we can further classify a large number of microviruses at the genus level and discover their evolutionary patterns. However, to ensure its accuracy, further investigations may be needed based on their genome structure and host. In conclusion, the DSV-related microviruses dataset and its phylogenetic analysis can further help explore new evolutionary pathways and evolutionary significance.

Cluster_4 (C4) generally exhibits a relatively tidy genome structure (Supplementary Figure S3). It is noteworthy that, despite being in the same cluster, there are significant differences in the host sources between DSV and NR viruses in C4. The majority of NR viruses are derived from wastewater metagenome samples, and their hosts are predominantly *Bacillus halmapalus*. *Bacillus halmapalus*, a halophilic bacterium, is a Gram-positive, alkaliphilic, alkalitolerant, facultative anaerobe. It is commonly isolated from soil, and its pathogenicity is not well understood (Nielsen et al., 1995). In DSV, only two viruses have *Bacillus halmapalus* as their host, and both are derived from sludge samples, aligning with the source of this bacterial species. Unlike NR, the primary hosts for DSV viruses are *Candidatus Pelagibacter ubique*. Except for Candidatus Pelagibacter microvirus C4_1, which originates from a swab of the transportation vehicle (CD-SXt), the rest are all from sludge samples (CD-SXD) (Supplementary Table S1). Studies suggest that *Candidatus Pelagibacter* genus may be among the most abundant bacteria globally and play a crucial role in the carbon cycle on Earth. *Croceibacter atlanticus* belongs to the phylum Bacteroidetes and is a species isolated from the Atlantic Ocean (Cho and Giovannoni, 2003). Croceibacter microvirus C4_1 is the only virus in this cluster derived from swab of duck oral and cloaca (D-SXAO) (Supplementary Table S1), and it is specifically associated with the host *Croceibacter atlanticus*. This observation once again confirms the conclusion from Figure 2E that the detection of microviruses is closely related to the distribution of their host bacteria and the source of the samples.

Cluster_5 (C5), as shown in Supplementary Figure S4. Although most NR sequences include Cap and Rep, DSV sequences, such as Bdellovibrio microvirus C5_2/4/7, Escherichia microvirus C5_1/2/13, only predict 2 ORFs: capsid protein and MinCP. We have not observed a correlation between this situation and sample sources, indicating a potentially higher novelty and lower conservation of genes in DSV sequences. Cluster_6 (C6) (Supplementary Figure S5), similar to C3 (Supplementary Figure S2), is a smaller cluster without predicted hosts, indicating a need for further research on this cluster. The genomic structure of the cluster_7 (C7) sequences is primarily arranged in the order of Cap, MinCP, SP, Rep, and DBP (Supplementary Figure S6). Croceibacter microvirus C7_1 and Croceibacter microvirus C7_2, two viruses within the same major branch, share *Croceibacter atlanticus* as their host (previously introduced in C4). This branch is the only one with predicted host results, while other branches lack host predictions. Therefore, C7 is also a potentially interesting virus cluster worthy of in-depth research. Cluster_8 (C8) (Supplementary Figure S7) has two distinct hosts, with *Ralstonia solanacearum* being the dominant host and *Achromobacter xylosoxidans* as the second host. *Ralstonia solanacearum* is considered one of the most important plant pathogens due to its lethal nature, persistence, wide host range, and extensive geographical distribution (Denny, 2006). *Achromobacter xylosoxidans* belongs to the genus *Achromobacter* and is commonly found in moist environments, causing diseases such as bacteremia, pneumonia, pharyngitis, and urinary tract infections (Igra-Siegman et al., 1980; Duggan et al., 1996).

### 3.5 Comparing DSV viruses in microvirus’s virosphere

**Figure 6:**
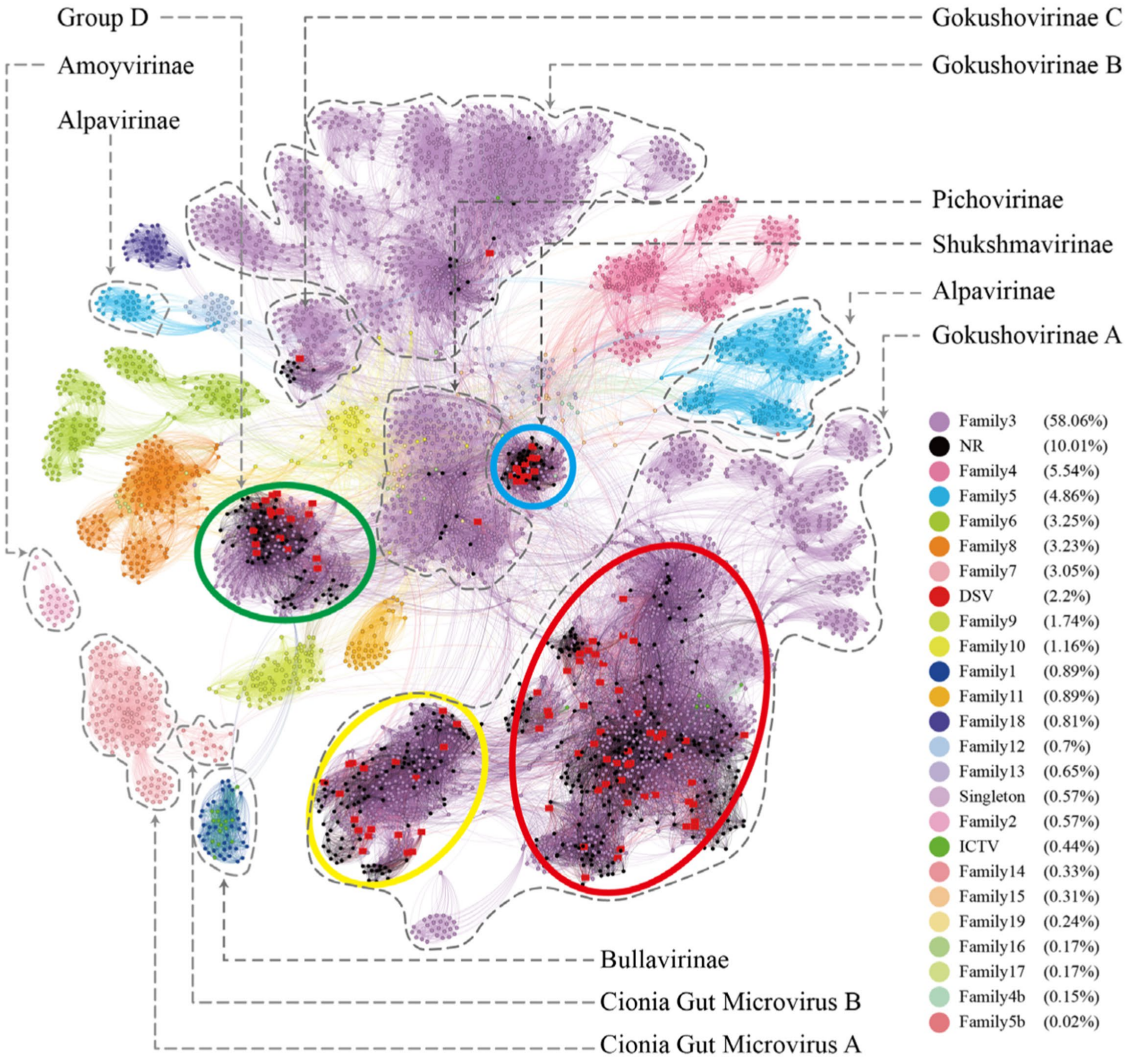
The microviruses collection with diverse taxa. Similarity clustering network was constructed using microviruses Cap sequences identified from DSV data (*n* = 98), along with related Cap sequences from NR (*n* = 459), ICTV microviruses Cap sequences (*n* = 20), and an additional set of Cap sequences reported by Kirchberger et al. (*n* = 4,007) [red dots represent DSV sequences, black dots represent NR sequences, and dark green dots represent ICTV sequences; other dots are colored based on the families defined by Kirchberger et al., 2022]. Clusters corresponding to those in Figure 1 are enclosed by ellipses of four different colors. Labels such as Pichovirinae, Shukshmavirinae, Group D, Alpavirinae, Gokushovirinae A/B/C correspond to subfamilies reported by previous studies (Krupovic and Forterre, 2011; Rosario et al., 2012; Roux et al., 2012; Tikhe and Husseneder, 2017) and suggested classifications by Kirchberger et al. (2022). The similarity clustering network graph was created using Gephi (version 0.9.7) based on Diamond (version 0.9.14.115) alignment score, with gray edges indicating Diamond Blastp score > 0.

To better understand the relationship between the identified microviruses in the poultry slaughterhouse and other reported microviruses in the Microviridae family, we expanded our focus beyond the 577 viral genomes highlighted in this study (Figure 1). To this end, an additional set of 4,077 microvirus Cap sequences (utilizing 4,007 sequences for this study) studied by Kirchberger et al. (2022) were incorporated into our analysis for a more comprehensive clustering analysis. In the study by Kirchberger et al., microviruses were broadly classified into 19 families, corresponding to the 19 color-coded clusters in Figure 6. Upon comparing the clustering results between Figures 1, 6, there is a good overall agreement between the two figures. Specifically, the four families identified in Figure 1 are concentrated within the purple cluster in Figure 6, representing the largest cluster in Family 3, as defined by Kirchberger et al. DSV viral sequences are predominantly distributed within Gokushovirinae A (Kirchberger et al., 2022), Shukshmavirinae (Tikhe and Husseneder, 2017) and Group D (Rosario et al., 2012) of Family 3. Additionally, three scattered sequences are found in Pichovirinae (Roux et al., 2012), Gokushovirinae B and Gokushovirinae C (Kirchberger et al., 2022), indicating that microviruses in the poultry slaughterhouse environment primarily belong to these groups. This result suggests that, although microviruses in the poultry slaughterhouse environment exhibit high diversity and novelty, they may still be relatively underrepresented in the entire microvirus virosphere. Family 3, possibly due to its close association with the human environment, is the largest group within the Microviridae. The expansion of other groups awaits further supplementation with samples from different sources and microbial hosts.

On the other hand, according to the clustering results in Figure 6, the 19 major families delineated by Kirchberger et al. can be further subdivided into approximately 45 smaller clusters. Particularly, within family 3 (the purple clusters), our clustering method can split it into as many as 20 smaller clusters. Specifically, Group D, Shukshmavirinae, Pichovirinae, and Gokushovirinae C each form independent cluster, while Gokushovirinae A and Gokushovirinae B can be further divided into multiple clusters at the same hierarchical level. These clusters are at least equivalent in status to Group D, Shukshmavirinae, and Pichovirinae. Additionally, families of other colors can also be further subdivided into smaller clusters. For example, family 5 identified as Alpavirinae (Krupovic and Forterre, 2011) can be distinctly clustered into 4 different clusters in this study. This suggests that these smaller clusters may represent novel subfamilies or families that require further identification.

### 3.6 The relationship between the clusters of microviruses and their host sources

**Figure 7:**
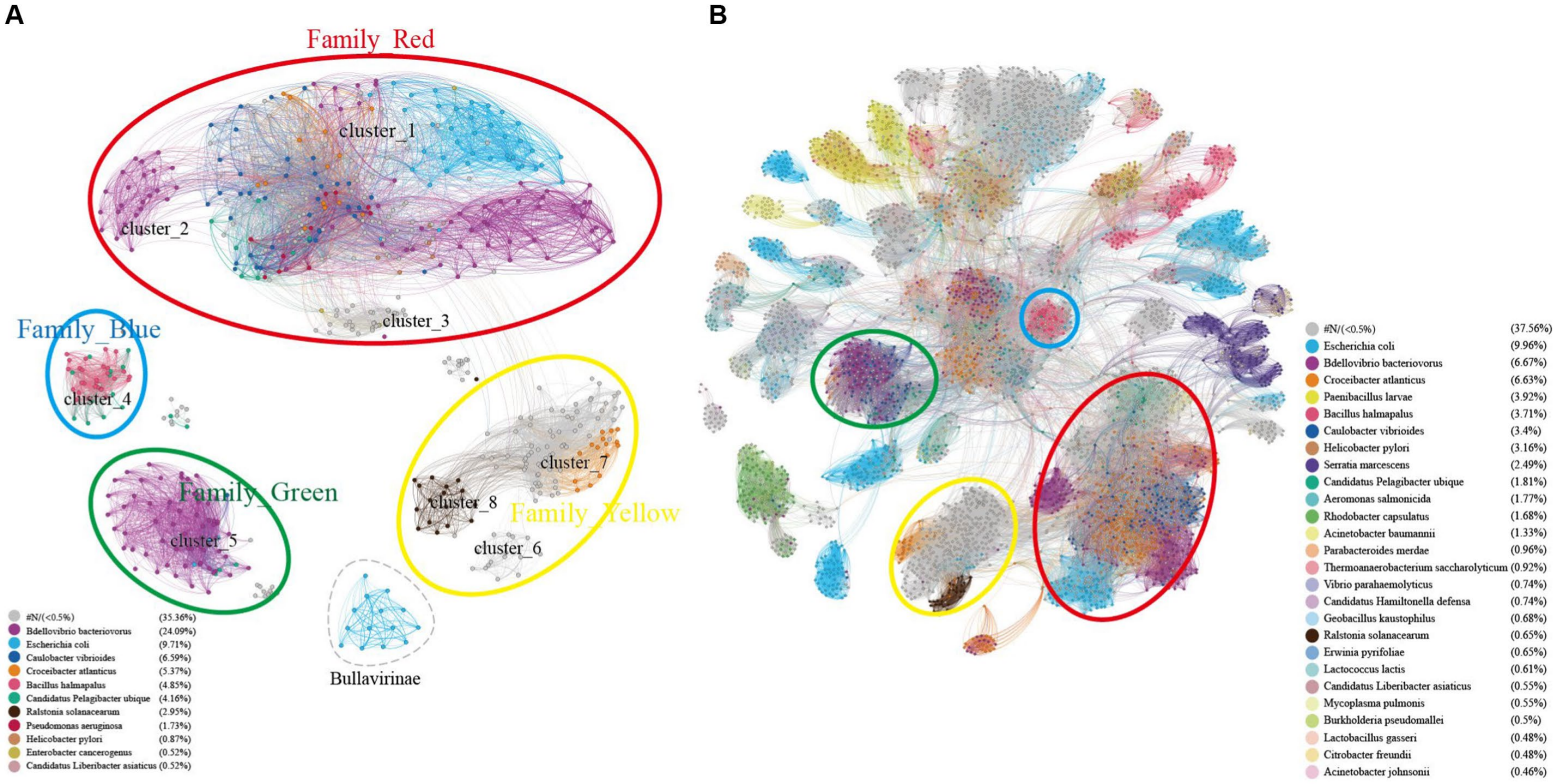
Host specificity of different clusters of Microviruses. **(A)** Similarity clustering network constructed using microviruses Cap sequences identified in DSV data (*n* = 98), along with related Cap sequences from NR (*n* = 459), and microviruses Cap sequences from ICTV (*n* = 20), colored based on host types. **(B)** Based on the sequences in panel **(A)**, an extended similarity clustering network was constructed by introducing Cap sequences reported by Kirchberssger et al. (*n* = 4,007), also colored according to host types. The similarity clustering network graph was created using Gephi (version 0.9.7) based on Diamond (version 0.9.14.115) alignment score, with gray edges indicating Diamond Blastp score > 0.

According to the cherry (Shang and Sun, 2022) results, the points in Figures 1, 6 are colored coded on host types in Figure 7. As shown in Figure 7A, clusters C2, C4, C5, a portion of C7, C8, and the Bullavirinae cluster exhibit clear host specificity, while the host colors in cluster C1 are highly mixed. Specifically, *Bdellovibrio bacteriovorus* is mainly the host for C2 and C5, the hosts for C4 are primarily *Bacillus halmapalus* and *Candidatus Pelagibacter ubique*, the main host for C8 is *Ralstonia solanacearum*, and only some sequences in C7 have host results, all of which are associated with *Croceibacter atlanticus*. C1 includes a significant number of *Bdellovibrio bacteriovorus* viruses, as well as viruses infecting *Escherichia coli*, *Caulobacter vibrioides*, *Croceibacter atlanticus*, and other bacteria. This may suggest that this group of viruses is more prone to host jumping compared to other viruses group.

Similar to the results in Figure 7A, the host sources of Family_Red members in Figure 7B remain diverse, primarily involving *Bdellovibrio bacteriovorus*, *Caulobacter vibrioides*, *Croceibacter atlanticus*, *Escherichia coli*. As the number of members increases, Family_Green shows an expanded range of host types, mainly associated with *Caulobacter vibrioides*, *Bdellovibrio bacteriovorus*, and *Candidatus Pelagibacter ubique*. Family_Blue continues to be dominated by *Bacillus halmapalus* and *Candidatus Hamiltonella defensa*. Although the number of Family_Yellow members has increased significantly, a majority still lacks predicted hosts. Apart from the four main groups focused on in this study, the host types of most smaller groups are relatively singular, such as *Bacillus halmapalus* (family4), *Escherichia coli* (family5), *Rhodobacter capsulatus* (family7) (Figure 7B). These results suggest variations in host specificity among different viral groups. Moreover, the good correspondence between similarity clustering networks and host prediction results is evident.

## 4 Discussion

Viruses are the most abundant life forms on earth. It is estimated that there are as many as 10^31^ virus particles on earth (Geoghegan and Holmes, 2017; Mushegian, 2020). However, The International Committee on Taxonomy of Viruses (ICTV) has officially recognized only around 12,000 known virus species.^3^ Viruses are often considered the “dark matter” of life sciences. Due to the challenges in cultivating many viruses, our understanding is limited to those that are easily cultivated and have significant impacts on humans or the economy. Advances in high-throughput sequencing and virome technologies have overcome the dependency on host cell cultures in traditional virology research, greatly enhancing the efficiency of discovering and identifying new viruses (Chapagain, 2019). In recent years, virome technologies has been widely applied in various studies, including marine environments and research on vertebrates and invertebrates, leading to the identification of numerous novel viruses (Zhu et al., 2022; Jiang et al., 2023) and significantly expanding our knowledge of the viral world.

Microviridae is one of the most common families of single-stranded DNA (ssDNA) viruses. Compared to double-stranded DNA (dsDNA) phages, the genomes of Microviridae are smaller, typically exhibiting higher safety by being less prone to carry virulence and resistance genes (Kirchberger et al., 2022). Moreover, they are widely distributed across various ecosystems (Angly et al., 2009; López-Bueno et al., 2009), representing a relatively accessible and exploitable source of DNA resources. As of now, the ICTV recognizes only two subfamilies within Microviridae, namely Bullavirinae and Gokushovirinae. However, this classification does not fully capture the extensive diversity of newly reported microviruses (Kirchberger et al., 2022). In recent years, numerous new taxonomic groups within the Microviridae family have been proposed. For instance, Krupovic et al. first defined a new Microviridae subfamily - Alpavirinae, which represents prophages of two bacterial genera within the phylum Proteobacteria, as confirmed by metagenomic analyses (Krupovic and Forterre, 2011). Other newly proposed subfamilies of Microviridae include Pichovirinae, assembled from 81 new microviral genomes retrieved from public database viromes by Roux et al. (2012). In addition, there are the microviruses subfamilies Sukshmavirinae found in termite gut viromes (Tikhe and Husseneder, 2017), Group D from dragonfly viromes (Rosario et al., 2012), and Aravirinae and Stokavirinae discovered in sphagnum- dominated peatlands (Quaiser et al., 2015). Kirchberger et al. comprehensively analyzed the genomes of microviruses using their classification method, providing insights into the diversity, distribution, and host range of this viral group (Kirchberger et al., 2022). The proposed new classifications are clearly represented in the clustering network graph of this study (Figure 6), indicating a good validation across different research efforts.

As far as we know, this study represents a relatively comprehensive compilation of members of the Microviridae, providing an overview of the classification of Microviridae and holding significant importance for the identification, exploration, and expansion of Microviridae. However, due to the large number of potential new taxa, this study did not assign explicit taxonomic names to them, focusing instead on demonstrating relationships between clusters. We believe that as more members of Microviridae are discovered and identified, this family will continue to give rise to new taxa and may undergo redefinition. To address this situation, there is an urgent need for a universal and straightforward method for classification, such as utilizing numbers or letters to systematically name newly emerging taxa.

The evolutionary trajectory of dsDNA phages is primarily influenced by horizontal gene exchange, driving the diversity and adaptive evolution of this phage class. However, for ssDNA phages, the evolutionary patterns may fundamentally differ (Hendrix et al., 1999, 2000; Doore and Fane, 2016). For instance, in microviruses, gene recombination is not widespread, and the presence of Cap may limit the insertion of foreign DNA sequences (Brentlinger et al., 2002), potentially restricting gene transfer at the horizontal level. Despite these factors, microviruses exhibit high mutation rates in their genomes (Minot et al., 2013), suggesting that they may employ different evolutionary strategies to enhance adaptability. This adaptability is evident in the diverse clusters and extensive host range discovered in this study, as well as the diversity in hosts and genome structures found even within the same phylogenetic branch. Doore and Fane (2016) conducted a comprehensive study on the structure and evolution of microviruses, revealing genomic structural differences among different genera of microviruses. As observed in this study and other studies (Roux et al., 2012), there are also certain differences in the genome structure of microviruses from different sample sources or types. This reflects the complexity and diversity of their evolution, showcasing their ability to adapt to different environments.

In this study, the sequences of microviruses were more readily detected in soil and sludge, which is consistent with previous reports on the occurrence of microviruses (Quaiser et al., 2015; Kirchberger et al., 2022). However, this is understandable as both bacteria and viruses in soil and sludge are relatively abundant. The lower detection rate of microviruses in other samples may also suggest that the types and abundance of bacteria and viruses in these sample types are lower or the abundance of microviruses’ hosts is lower. But the abundance of microviruses may not accurately represent the abundance of various microorganisms in other sample types. In addition, we consider that MDA amplification has already selectively amplified ssDNA genomes, the abundance data may not be accurate. On the other hand, virus and host abundances are often correlated. Although our study has identified many potential new hosts, we have not yet verified the abundance of these hosts or even their existence. Therefore, we believe that analyzing virus abundance solely based on sequencing data may not be very meaningful or accurate. Further exploration of the relative abundance of these sequences in the entire genome sequence will require more work and deeper research. We hope to experimentally study correlation between bacterial abundance and virus abundance in future research and test this point.

At present, the mainstream view suggests that the hosts of microviruses are primarily intracellular parasitic bacteria and Enterobacteria. For instance, the host of the Bullavirinae is Enterobacteria, and detailed studies have been conducted on representatives of this family, such as the phage ΦX174 (Doore and Fane, 2016). Members of the Gokushovirinae only infect *Chlamydia*, *Bdellovibrio* and *Spiroplasma* (Brentlinger et al., 2002; King et al., 2012). However, an increasing number of studies indicate that microviruses can infect a broader range of bacterial hosts, including *Vibrio parahaemolyticus* (Guo et al., 2022; Xu et al., 2022), *Salmonella* (Li et al., 2021), *Shigella flexneri* (Lu et al., 2022) and other bacteria. Predicting the hosts of these viruses is challenging because many cellular organisms lack genomic data or have not been extensively studied for their viruses. However, Host Prediction and Phylogenetic Analysis provide feasible solutions for predicting the hosts of these viruses. On the one hand, machine learning tools like Cherry can identify key features, improving the accuracy and scope of virus host prediction. On the other hand, members of closely related virus families tend to infect similar hosts, providing strong support for expanding the host range of Microviridae. This study employed two new host prediction methods, the hostG utilizes shared protein clusters between viruses and prokaryotes to create a knowledge graph and trains a graph convolutional network for prediction (Shang and Sun, 2021). While it achieves high prediction accuracy, its results tend to be conservative and can only predict hosts at the genus level. Cherry is described as having the highest accuracy in identifying virus-prokaryote interactions, outperforming all existing methods at the species level, with an accuracy of 80% (Shang and Sun, 2022). The results from Cherry indicate that the hosts of the Microviridae exhibit extremely high diversity, including various pathogens such as *Mycoplasma pulmonis*, *Helicobacter pylori*, *Vibrio cholerae*, *Clostridioides difficile*, and *Pseudomonas aeruginosa*. Additionally, this study identified some plant-pathogenic bacteria, such as *Ralstonia solanacearum* (*R. solanacearum*) and *Candidatus Liberibacter asiaticus* (*CLas*). Bacterial wilt, caused by *R. solanacearum*, is economically significant as it can infect over 250 plant species, including potatoes, tomatoes, and tobacco, causing substantial yield losses in tropical and subtropical regions (Wicker et al., 2007; Champoiseau et al., 2009). *CLas* is the pathogen responsible for citrus Huanglongbing (HLB, also known as citrus greening disease) (Wang et al., 2022), a highly destructive disease threatening global citrus production. There has been limited research on Microviridae infecting plant-pathogenic bacteria, and the findings of this study suggest that Microviridae also holds potential for applications in the control of bacterial diseases in plants.

It should be noted that the discovery of numerous potential hosts for microviruses is not surprising, given the large number and widespread distribution of microviruses, implying foreseeable diversity in their types and hosts. Moreover, machine learning-based host prediction tools like hostG and cherry can identify more hidden genomic information, providing more reliable prediction results. Additionally, the sources of hosts for microviruses exhibit certain regularities and complexities. As depicted in the host network graph (Figure 7), some clusters show strong host specificity, while others are more prone to host crossing. With the discovery of numerous new hosts and understanding their cluster distribution, we can easily explore the evolution and cluster characteristics of microviruses. In summary, a comprehensive understanding of the host range of microviruses helps uncover more information about this viral family and their ecological functions and potential biological impacts in the environment. Furthermore, understanding the interaction between microviruses and the environment contributes to a better understanding of the transmission routes and pathogenic mechanisms of microbial pathogens.

## 5 Conclusion

This study employed virome techniques to thoroughly explore potential members of Microviridae in a poultry slaughterhouse, successfully identifying and analyzing 98 novel and complete microvirus genomes. Based on the similarity of Cap proteins, it was discovered that these genomes represent at least six new subfamilies within Microviridae, distinct from Bullavirinae and Gokushovirinae, as well as three higher-level classification units. These new taxa exhibited evident regularities in genome size, GC content, and genome structure, further highlighting the rationality of the classification method used in this study. Additionally, based on the 19 families classified by previous researchers for all microviruses, our results divided microviruses into about 45 more detailed clusters, which may serve as a new standard for classifying Microviridae members. The current information on microviruses’ hosts remains limited, and this study significantly expands their host range. In addition to typical hosts such as intracellular parasitic bacteria and Enterobacteria, we identified over 20 potential new hosts, including important pathogens such as *Helicobacter pylori* and *Vibrio cholerae*. Moreover, our data revealed distinct host specific differences among different taxa. These new findings will contribute to a deeper understanding of the interactions between Microviridae and their hosts.

## Data availability statement

The dataset supporting the results of this article has been deposited in the National Center for Biotechnology Information (NCBI) under BioProject accession code PRJNA1053868. All viral genomes obtained in this study were deposited in GenBank with the accession numbers: OR998966-9063.

## Ethics statement

This study involved in the collection of human nasal swabs and animal swabs and was approved by Medical Ethics Committee of the School of Public Health, Sun Yat-sen University (2018-001). The study was conducted in accordance with the local legislation and institutional requirements. The participants provided their written informed consent to participate in this study.

## Author contributions

KX: Methodology, Data curation, Formal analysis, Visualization, Writing – original draft, Validation. BL: Methodology, Conceptualization, Funding acquisition, Investigation, Project administration, Writing – review & editing. XS: Investigation, Data curation, Writing – original draft. PZ: Data curation, Investigation, Writing – original draft. CL: Data curation, Investigation, Writing – original draft. GL: Data curation, Investigation, Writing – original draft. XC: Writing – review & editing. JP: Investigation, Writing – original draft. SQ: Investigation, Writing – original draft. XY: Investigation, Writing – original draft. ML: Investigation, Writing – original draft. JJ: Writing – original draft, Writing – review & editing, Conceptualization, Funding acquisition, Methodology, Project administration, Supervision. LY: Conceptualization, Funding acquisition, Methodology, Project administration, Resources, Supervision, Writing – review & editing.

## Footnotes

^1^www.snapgene.com, version 4.3.6.

^2^ https://itol.embl.de

^3^https://ictv.global/vmr, Version: VMR MSL38 v1.

## Supplementary material

SUPPLEMENTARY FIGURE S1 Phylogenetic tree, hosts, and genomic structure of cluster_1 microviruses from poultry slaughterhouse and related sources.

SUPPLEMENTARY FIGURE S2 Phylogenetic tree, hosts, and genomic structure of cluster_3 microviruses from poultry slaughterhouse and related sources.

SUPPLEMENTARY FIGURE S3 Phylogenetic tree, hosts, and genomic structure of cluster_4 microviruses from poultry slaughterhouse and related sources.

SUPPLEMENTARY FIGURE S4 Phylogenetic tree, hosts, and genomic structure of cluster_5 microviruses from poultry slaughterhouse and related sources.

SUPPLEMENTARY FIGURE S5 Phylogenetic tree, hosts, and genomic structure of cluster_6 microviruses from poultry slaughterhouse and related sources.

SUPPLEMENTARY FIGURE S6 Phylogenetic tree, hosts, and genomic structure of cluster_7 microviruses from poultry slaughterhouse and related sources.

SUPPLEMENTARY FIGURE S7 Phylogenetic tree, hosts, and genomic structure of cluster_8 microviruses from poultry slaughterhouse and related sources.

SUPPLEMENTARY TABLE S1 Genomic sequence information of poultry slaughterhouse associated microviruses.

SUPPLEMENTARY TABLE S2 Genomic sequence information of poultry slaughterhouse associated microviruses.

SUPPLEMENTARY TABLE S3 Microviruses Cap from NCBI NR database.
