# Supplementary figures and images for: Identification and classification of the genomes of novel microviruses in poultry slaughterhouse
Source: Front Microbiol. 2024 May 2;15:1393153. doi: 10.3389/fmicb.2024.1393153 (PMC11096546; doi:10.3389/fmicb.2024.1393153)

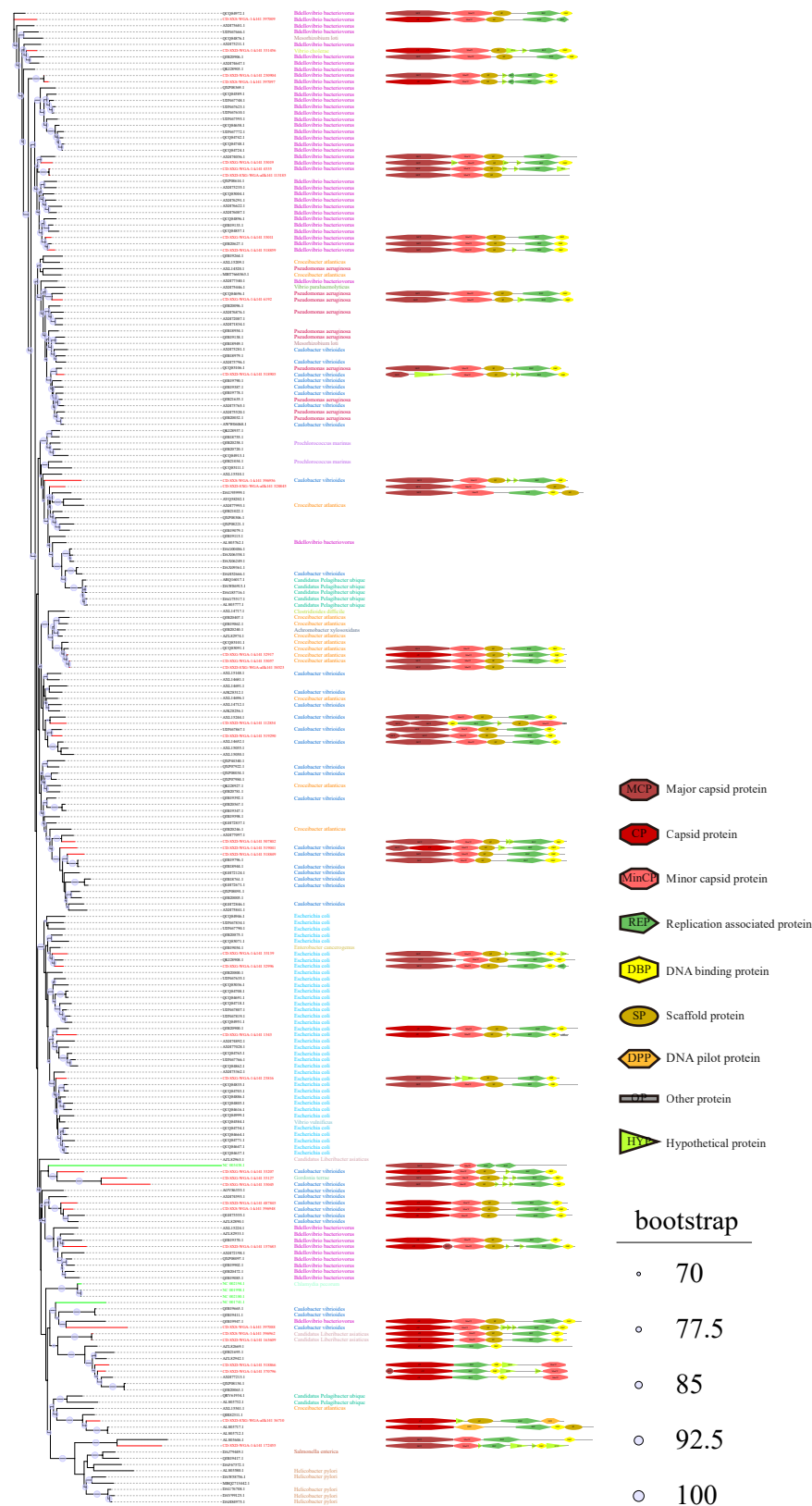

Supplement: SUPPLEMENTARY FIGURE S1 — Phylogenetic tree, hosts, and genomic structure of cluster_1 microviruses from poultry slaughterhouse and related sources. [file Image_1.PDF]

Tree scale: 1

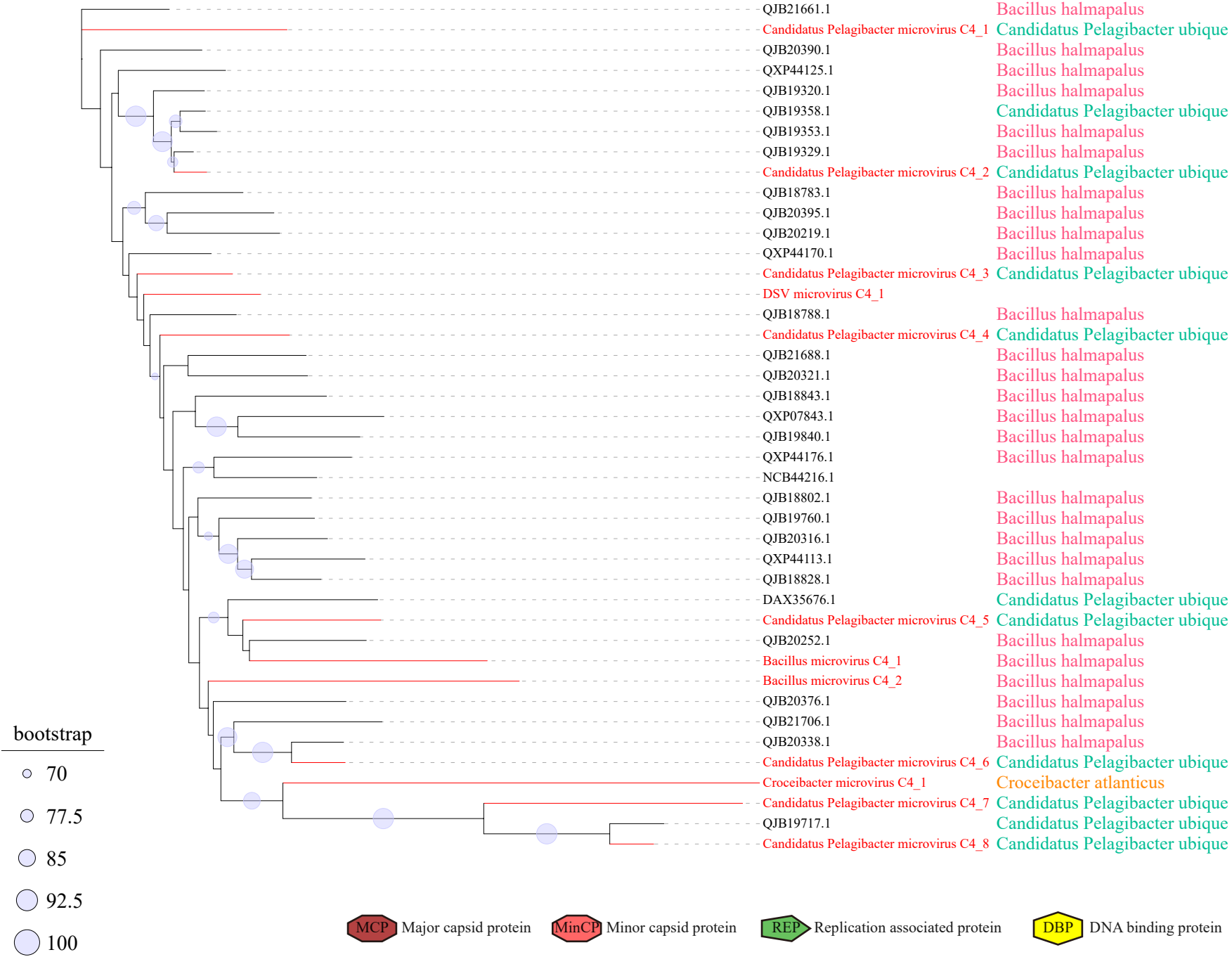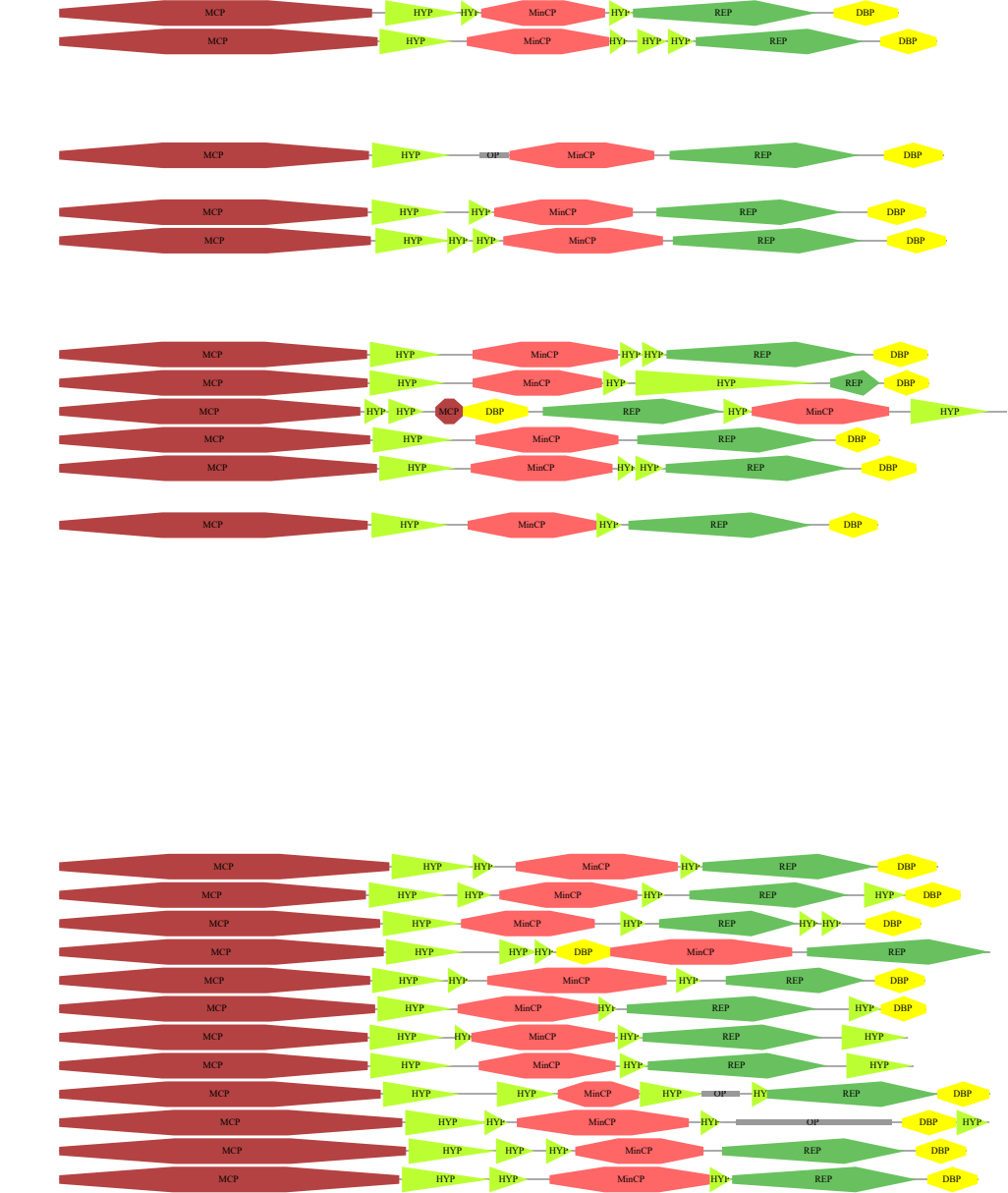

Supplement: SUPPLEMENTARY FIGURE S3 — Phylogenetic tree, hosts, and genomic structure of cluster_4 microviruses from poultry slaughterhouse and related sources. [file Image_3.PDF]

Tree scale: 0.01

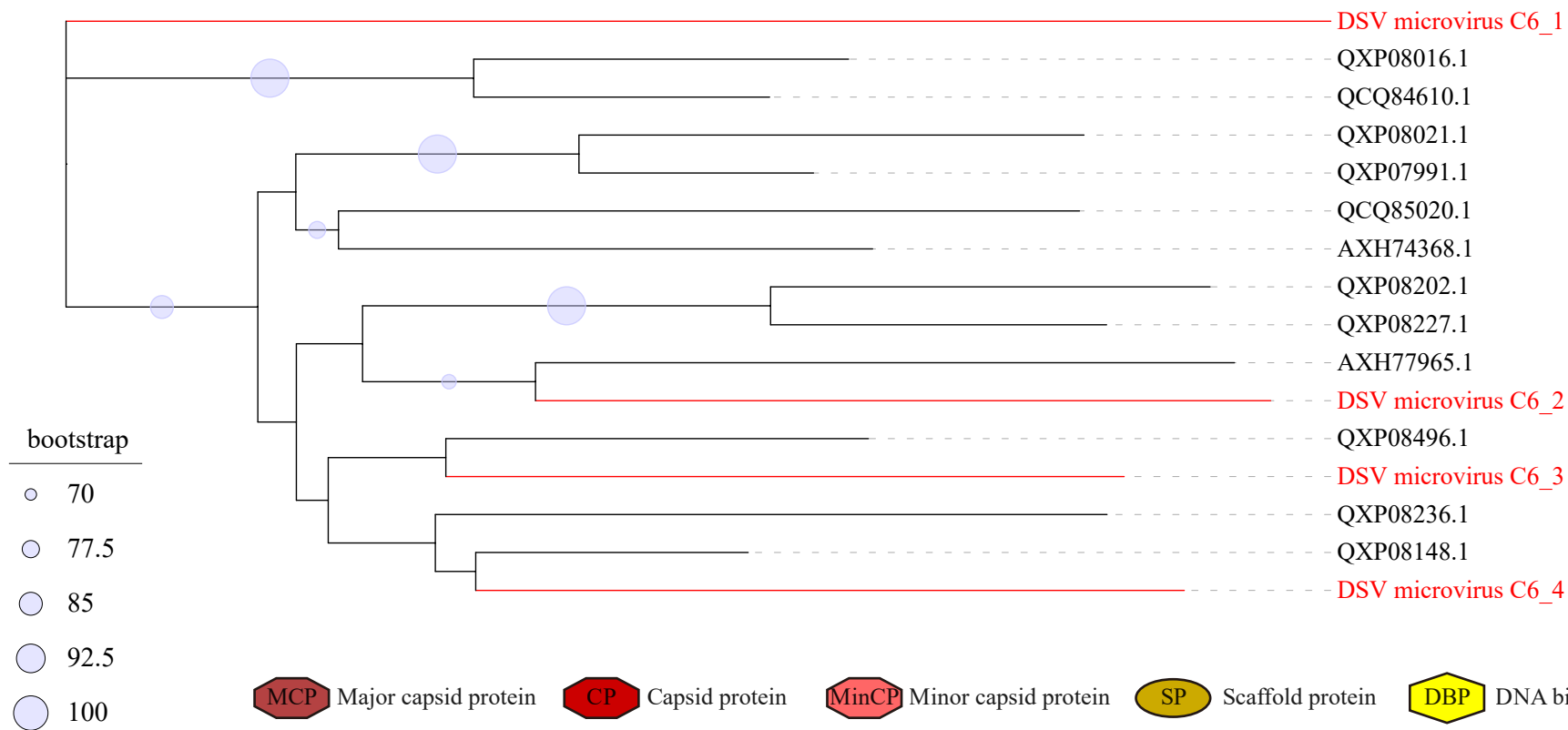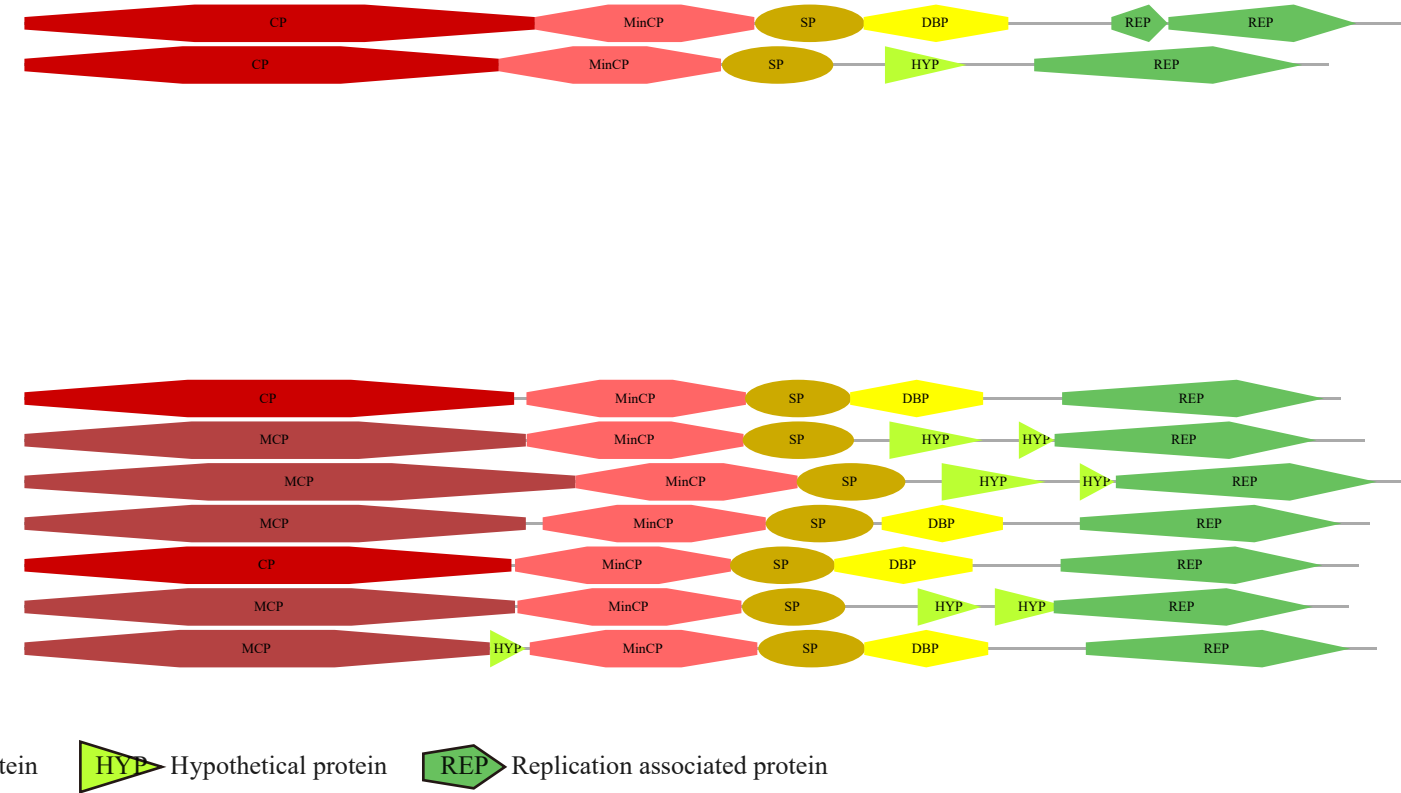

Supplement: SUPPLEMENTARY FIGURE S5 — Phylogenetic tree, hosts, and genomic structure of cluster_6 microviruses from poultry slaughterhouse and related sources. [file Image_5.PDF]

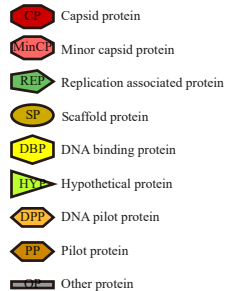

Supplement: SUPPLEMENTARY FIGURE S6 — Phylogenetic tree, hosts, and genomic structure of cluster_7 microviruses from poultry slaughterhouse and related sources. [file Image_6.PDF]

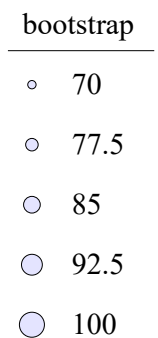

Supplement: SUPPLEMENTARY FIGURE S7 — Phylogenetic tree, hosts, and genomic structure of cluster_8 microviruses from poultry slaughterhouse and related sources. [file Image_7.PDF]
