# Supplementary material for: Identification and classification of the genomes of novel microviruses in poultry slaughterhouse
Source: Front Microbiol. 2024 May 2;15:1393153. doi: 10.3389/fmicb.2024.1393153 (PMC11096546; doi:10.3389/fmicb.2024.1393153)
Supplement: SUPPLEMENTARY FIGURE S2 — Phylogenetic tree, hosts, and genomic structure of cluster_3 microviruses from poultry slaughterhouse and related sources. [file Image_2.PDF]

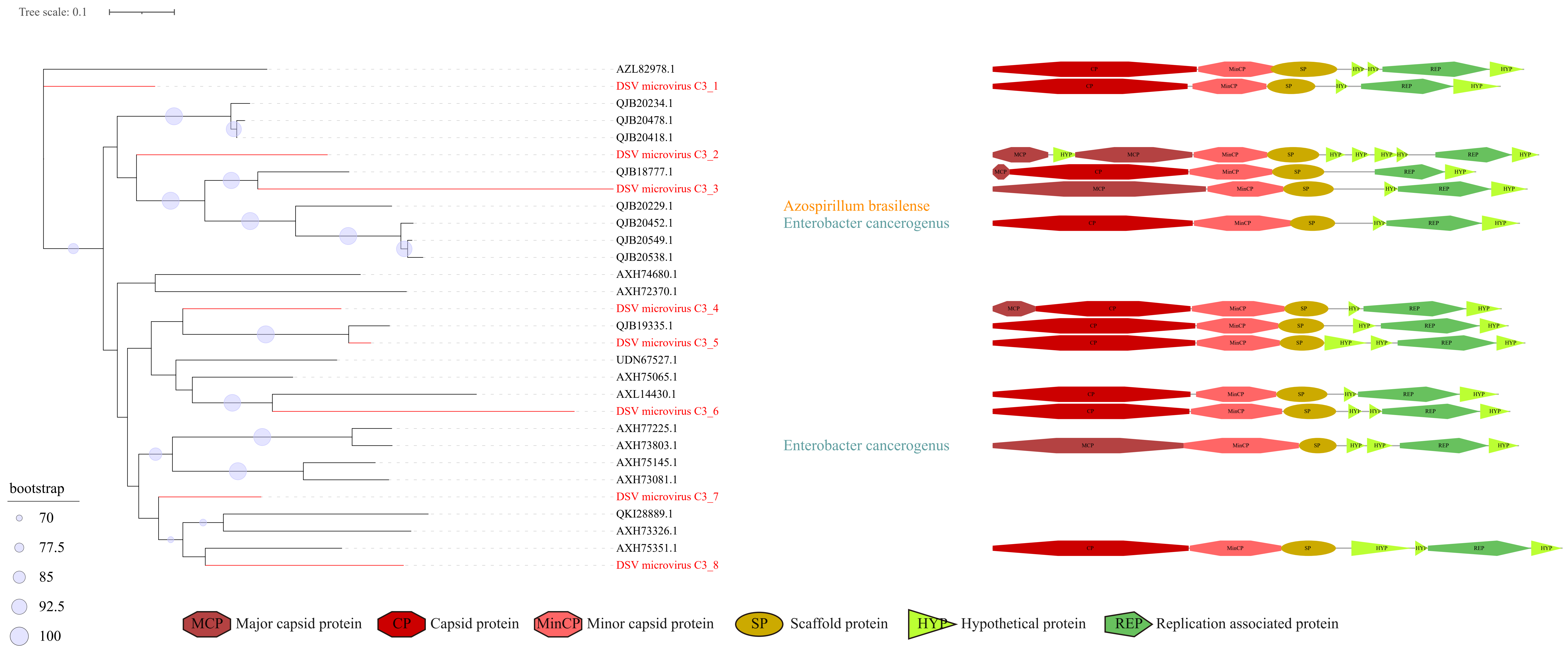
Tree scale: 0.1
AZL82978.1
DSV microvirus C3_1
QJB20234.1
QJB20478.1
QJB20418.1
DSV microvirus C3_2
QJB18777.1
DSV microvirus C3_3
QJB20229.1
QJB20452.1
QJB20549.1
QJB20538.1
AXH74680.1
AXH72370.1
DSV microvirus C3_4
QJB19335.1
DSV microvirus C3_5
UDN67527.1
AXH75065.1
AXL14430.1
DSV microvirus C3_6
AXH77225.1
AXH73803.1
AXH75145.1
AXH73081.1
DSV microvirus C3_7
QKI28889.1
AXH73326.1
AXH75351.1
DSV microvirus C3_8
Azospirillum brasilense
Enterobacter cancerogenus
Enterobacter cancerogenus
bootstrap
70
77.5
85
92.5
100
MCP Major capsid protein
CP Capsid protein
MinCP Minor capsid protein
SP Scaffold protein
HYP Hypothetical protein
REP Replication associated protein
