# Supplementary material for: Identification and classification of the genomes of novel microviruses in poultry slaughterhouse
Source: Front Microbiol. 2024 May 2;15:1393153. doi: 10.3389/fmicb.2024.1393153 (PMC11096546; doi:10.3389/fmicb.2024.1393153)
Supplement: SUPPLEMENTARY FIGURE S4 — Phylogenetic tree, hosts, and genomic structure of cluster_5 microviruses from poultry slaughterhouse and related sources. [file Image_4.PDF]

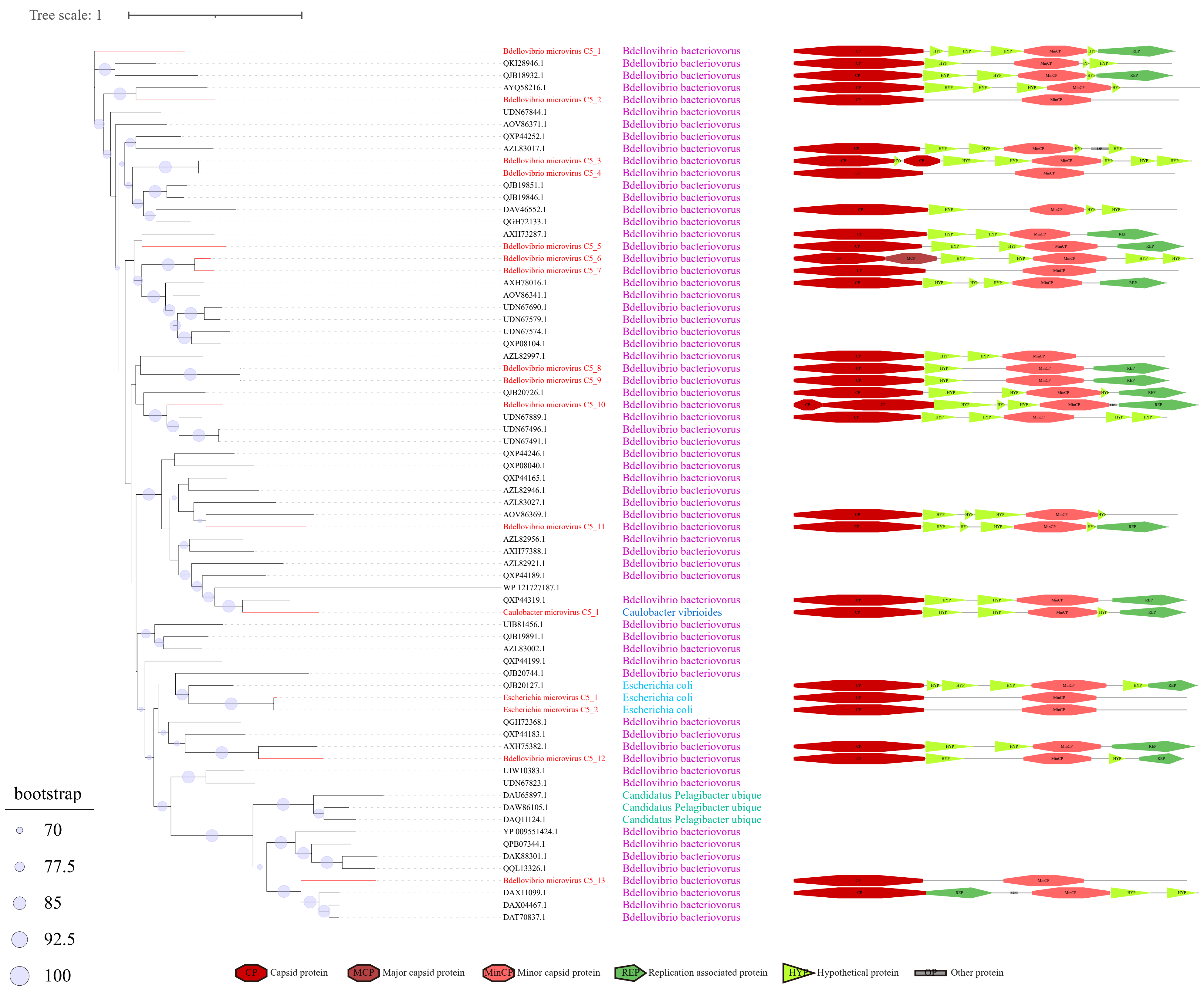

Tree scale: 1
Bdellovibrio microvirus C5_1 Bdellovibrio bacteriovorus
QKI28946.1 Bdellovibrio bacteriovorus
QJB18932.1 Bdellovibrio bacteriovorus
AYQ58216.1 Bdellovibrio bacteriovorus
Bdellovibrio microvirus C5_2 Bdellovibrio bacteriovorus
UDN67844.1 Bdellovibrio bacteriovorus
AOV86371.1 Bdellovibrio bacteriovorus
QXP44252.1 Bdellovibrio bacteriovorus
AZL83017.1 Bdellovibrio bacteriovorus
Bdellovibrio microvirus C5_3 Bdellovibrio bacteriovorus
Bdellovibrio microvirus C5_4 Bdellovibrio bacteriovorus
QJB19851.1 Bdellovibrio bacteriovorus
QJB19846.1 Bdellovibrio bacteriovorus
DAV46552.1 Bdellovibrio bacteriovorus
QGH72133.1 Bdellovibrio bacteriovorus
AXH73287.1 Bdellovibrio bacteriovorus
Bdellovibrio microvirus C5_5 Bdellovibrio bacteriovorus
Bdellovibrio microvirus C5_6 Bdellovibrio bacteriovorus
Bdellovibrio microvirus C5_7 Bdellovibrio bacteriovorus
AXH78016.1 Bdellovibrio bacteriovorus
AOV86341.1 Bdellovibrio bacteriovorus
UDN67690.1 Bdellovibrio bacteriovorus
UDN67579.1 Bdellovibrio bacteriovorus
UDN67574.1 Bdellovibrio bacteriovorus
QXP08104.1 Bdellovibrio bacteriovorus
AZL82997.1 Bdellovibrio bacteriovorus
Bdellovibrio microvirus C5_8 Bdellovibrio bacteriovorus
Bdellovibrio microvirus C5_9 Bdellovibrio bacteriovorus
QJB20726.1 Bdellovibrio bacteriovorus
Bdellovibrio microvirus C5_10 Bdellovibrio bacteriovorus
UDN67889.1 Bdellovibrio bacteriovorus
UDN67496.1 Bdellovibrio bacteriovorus
UDN67491.1 Bdellovibrio bacteriovorus
QXP44246.1 Bdellovibrio bacteriovorus
QXP08040.1 Bdellovibrio bacteriovorus
QXP44165.1 Bdellovibrio bacteriovorus
AZL82946.1 Bdellovibrio bacteriovorus
AZL83027.1 Bdellovibrio bacteriovorus
AOV86369.1 Bdellovibrio bacteriovorus
Bdellovibrio microvirus C5_11 Bdellovibrio bacteriovorus
AZL82956.1 Bdellovibrio bacteriovorus
AXH77388.1 Bdellovibrio bacteriovorus
AZL82921.1 Bdellovibrio bacteriovorus
QXP44189.1 Bdellovibrio bacteriovorus
WP 121727187.1
QXP44319.1 Bdellovibrio bacteriovorus
Caulobacter microvirus C5_1 Caulobacter vibrioides
UIB81456.1 Bdellovibrio bacteriovorus
QJB19891.1 Bdellovibrio bacteriovorus
AZL83002.1 Bdellovibrio bacteriovorus
QXP44199.1 Bdellovibrio bacteriovorus
QJB20744.1 Bdellovibrio bacteriovorus
QJB20127.1 Escherichia coli
Escherichia microvirus C5_1 Escherichia coli
Escherichia microvirus C5_2 Escherichia coli
QGH72368.1 Bdellovibrio bacteriovorus
QXP44183.1 Bdellovibrio bacteriovorus
AXH75382.1 Bdellovibrio bacteriovorus
Bdellovibrio microvirus C5_12 Bdellovibrio bacteriovorus
UIW10383.1 Bdellovibrio bacteriovorus
UDN67823.1 Bdellovibrio bacteriovorus
DAU65897.1 Candidatus Pelagibacter ubique
DAW86105.1 Candidatus Pelagibacter ubique
DAQ11124.1 Candidatus Pelagibacter ubique
YP 009551424.1 Bdellovibrio bacteriovorus
QPB07344.1 Bdellovibrio bacteriovorus
DAK88301.1 Bdellovibrio bacteriovorus
QQL13326.1 Bdellovibrio bacteriovorus
Bdellovibrio microvirus C5_13 Bdellovibrio bacteriovorus
DAX11099.1 Bdellovibrio bacteriovorus
DAX04467.1 Bdellovibrio bacteriovorus
DAT70837.1 Bdellovibrio bacteriovorus
bootstrap
70
77.5
85
92.5
100
CP Capsid protein
MCP Major capsid protein
MinCP Minor capsid protein
REP Replication associated protein
HYP Hypothetical protein
OP Other protein
